# Photocatalytic Hydrogen Production from Glycerol Aqueous Solutions as Sustainable Feedstocks Using Zr-Based UiO-66 Materials under Simulated Sunlight Irradiation

**DOI:** 10.3390/nano12213808

**Published:** 2022-10-28

**Authors:** Celia M. Rueda-Navarro, Belén Ferrer, Herme G. Baldoví, Sergio Navalón

**Affiliations:** Departamento de Química, Universitat Politècnica de València, C/Camino de Vera s/n, 46022 Valencia, Spain

**Keywords:** heterogeneous photocatalysis, hydrogen generation, metal–organic frameworks, glycerol feedstock, simulated sunlight irradiation

## Abstract

There is an increasing interest in developing cost-effective technologies to produce hydrogen from sustainable resources. Herein we show a comprehensive study on the use of metal–organic frameworks (MOFs) as heterogeneous photocatalysts for H_2_ generation from photoreforming of glycerol aqueous solutions under simulated sunlight irradiation. The list of materials employed in this study include some of the benchmark Zr-MOFs such as UiO-66(Zr)-X (X: H, NO_2_, NH_2_) as well as MIL-125(Ti)-NH_2_ as the reference Ti-MOF. Among these solids, UiO-66(Zr)-NH_2_ exhibits the highest photocatalytic H_2_ production, and this observation is attributed to its adequate energy level. The photocatalytic activity of UiO-66(Zr)-NH_2_ can be increased by deposition of small Pt NPs as the reference noble metal co-catalyst within the MOF network. This photocatalyst is effectively used for H_2_ generation at least for 70 h without loss of activity. The crystallinity of MOF and Pt particle size were maintained as revealed by powder X-ray diffraction and transmission electron microscopy measurements, respectively. Evidence in support of the occurrence of photoinduced charge separation with Pt@UiO-66(Zr)-NH_2_ is provided from transient absorption and photoluminescence spectroscopies together with photocurrent measurements. This study exemplifies the possibility of using MOFs as photocatalysts for the solar-driven H_2_ generation using sustainable feedstocks.

## 1. Introduction

Fossil fuels are still employed to supply more than 80% of the world energy demand [1]. The combustion of fossil fuels is directly connected to air pollution, emissions of greenhouse gases and climate change, resulting in negative impacts for the environment and human health [1,2,3]. For these reasons, there is an urgent need to develop alternative sustainable and renewable energy vectors that favor the decarbonization of the current energy system [4,5]. In this context, hydrogen is recognized as an ideal carbon-free energy vector candidate [6,7,8]. Today, however, hydrogen is mostly produced via steam reforming of hydrocarbons at high temperature (>650–1000 °C), with most energy requirements from nonrenewable fossil fuels [6,9]. Therefore, important scientific and technological efforts are being made to produce H_2_ in a sustainable and renewable manner [7,8]. Electrolysis processes are relatively efficient and mature technologies for decomposing H_2_O into H_2_ and O_2_ that can operate using, for example, renewable photovoltaic energy [10]. The economic viability of this technology is, however, still hampered due to the investment and operation costs [7,8,11,12]. Alternatively, solar-driven photocatalytic water splitting into H_2_ and O_2_ using heterogeneous catalysts is a simpler and cheaper technology with potential application at a medium–large scale [13,14,15]. For this purpose, it would be necessary further increase the relatively low efficiencies achieved using inorganic semiconductors or investigate other type of photocatalysts [13,15,16].

**Figure 1:**
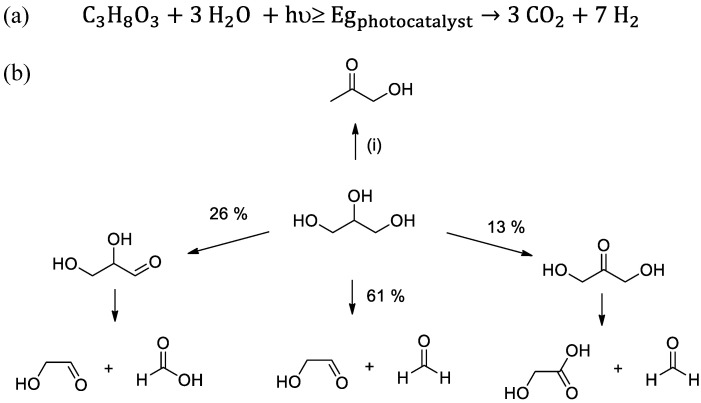
(**a**) Stoichiometry of glycerol photoreforming. (**b**) Proposed initial reaction sequences of glycerol photoreforming using Rh/TiO_2_. Note: Light-driven dehydration to hydroxyacetone constitutes a side reaction (i) [46].

Today, metal–organic frameworks (MOFs) [17,18,19] are considered among the most versatile and tunable materials to act as active photocatalysts [20,21,22,23]. Photocatalytic overall water splitting using MOFs is still a challenging process that is in its infancy [24,25,26]. A more efficient though less ambitious process closer to real small-to-medium industrial applications is the photocatalytic hydrogen evolution reaction (HER) in the presence of sacrificial electron donors [27]. The research in the field of photocatalytic HER using MOFs is exceptional, and excellent achievements have been reached even under visible or sunlight irradiation [27,28,29,30,31,32,33,34,35,36,37,38,39,40]. Most of the reports in this area have employed sacrificial electron donors such as methanol, triethanolamine (TEOA) or triethylamine to enhance the H_2_ production [27,28,29,30,31,32,33,34,35,36,37,38,39,40]. The use of these sacrificial agents limits, however, the cost-effective real application. Alternatively, in a similar way to that reported for inorganic semiconductors [27,41], the use of biomass or industrial byproduct feedstocks for H_2_ production is envisioned as a promising solution [42,43]. In this context, it is worth mentioning that biodiesel production generates about a 10 *w*/*w* % of crude glycerol as byproduct [44]. In this scenario, the development of technologies such as photoreforming of glycerol aqueous solution (Figure 1) will contribute to both the viability of the biodiesel market and the production of sustainable H_2_ [42,44]. Theoretically, complete glycerol photoreforming results in a H_2_-to-CO_2_ molar ratio of 2.33 (Figure 1a). The proposed reaction mechanism of glycerol photoreforming mainly using inorganic semiconductors is complicated and still under investigation [45]. Some studies have proposed that the oxidation process could proceed through different parallel reaction pathways involving initial oxidative C-C scission, oxidation of primary or secondary carbons (carbonyl group formation) or light-driven dehydration (Figure 1b) [46]. Importantly, the reaction pathway determines the H_2_-to-CO_2_ molar ratio [46]. The higher extent of carbonyl group formation with respect to oxidative C-C rupture leads to the higher H_2_:CO_2_ molar ratio [47]. Some studies have also reported the formation of byproducts such as formaldehyde, methane or CO among other possible products during glycerol photoreforming [45]. The occurrence of other processes such as photocatalytic water splitting might also take place simultaneously [24]. In the area of MOF-based materials, as far as we know, there is only one example reporting the use of an inorganic semiconductor/MOF composite, termed as TiO_2_@HKUST-1, used for hydrogen production from photoreforming of glycerol aqueous solutions (Figure 1a) [48].

With these precedents in mind, the present study investigated in a comprehensive manner the H_2_ generation from photoreforming of glycerol aqueous solution using Zr-based UiO-66 materials under simulated sunlight irradiation. Particularly, three UiO-66(Zr)-X (X: H, NH_2_, NO_2_) solids having different energy band levels were selected for the study. For comparison, the photoactivity of a Ti-based MOF reference material such as MIL-125(Ti)-NH_2_ was tested. The most active UiO-66(Zr)-NH_2_ photocatalyst of the series was further modified with Pt NPs as the noble metal reference co-catalyst. The photocatalyst activity and stability of the Pt@UiO-66(Zr)-NH_2_ was also studied. The reaction mechanism of the most active sample was further studied by means of transition absorption and photoluminescence spectroscopies as well as by photocurrent measurements.

## 2. Materials and Methods

### 2.1. Materials

All the chemicals employed in this work were of analytical or HPLC grade and supplied by Merck. 

### 2.2. Synthesis and Characterization of the MOF-Based Materials

The MOFs under study, namely, UiO-66(Zr), UiO-66(Zr)-NH_2_, UiO-66(Zr)-NO_2_ and MIL-125(Ti)-NH_2_, were prepared according to previously reported procedures [49,50]. Pt NPs (1 wt%) were supported on UiO-66(Zr)-NH_2_ by using the photodeposition method. The series of materials were characterized by several techniques including powder X-ray diffraction (PXRD), UV-Vis spectroscopy, X-ray photoelectron spectroscopy (XPS), thermogravimetric analysis (TGA), isothermal N_2_ adsorption, and scanning (SEM) and transmission (TEM) electron microscopies equipped with an EDX detector. The most active Pt@UiO-66(Zr)-NH_2_ and/or UiO-66(Zr)-NH_2_ photocatalysts were also characterized by means of transient absorption (TAS) and photoluminescence (PL) spectroscopies and photocurrent measurements. Supporting information summarizes the details of these experimental procedures and the characterization techniques employed in this study.

### 2.3. Photocatalytic Hydrogen Generation from Glycerol Aqueous Solution

Briefly, 5 mg of MOF photocatalyst was placed in a quartz reactor (51 mL) containing a glycerol aqueous mixture (20 mL), and then the system was sonicated (450 W) for 20 min to obtain a good MOF dispersion. To remove the air from the reactor, the system was purged with argon for 1 h. The MOF suspension while stirring was irradiated under simulated sunlight irradiation (Hamamatsu Hg-Xe lamp-150W-L8253; Hamamatsu spotlight source-L9566-04; Hamamatsu light guide A10014-50-0110; Lasing AM 1.5 G type filter-81094). During the irradiation, the temperature and the pressure of the system were monitored. At the required time, the evolved gases were analyzed from the head space of the quartz reactor by direct connection to a Micro GC system (Agilent 490 Micro GC system equipped with a Molsieve 5 A° column) that employed argon as the carrier gas. These photocatalytic measurements were carried out at least in triplicate trials, and the presented data correspond to the average of these experiments.

## 3. Results and Discussion

### 3.1. Photocatalyst Preparation and Characterization

**Figure 2:**
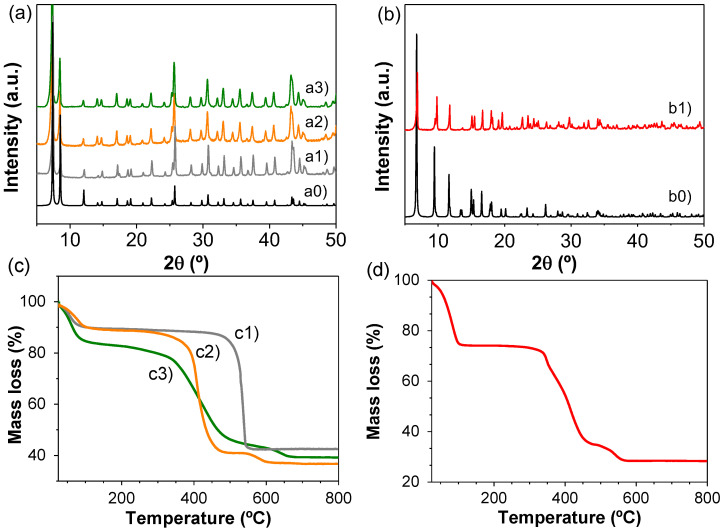
(**a**) XRD patterns of simulated UiO-66(Zr) (a0) and experimental UiO-66(Zr) (a1), UiO-66(Zr)-NO_2_ (a2) and UiO-66(Zr)-NH_2_ (a3). (**b**) XRD patterns of simulated (b0) and experimental MIL-125(Ti)-NH_2_ (b1). (**c**) TGA of UiO-66(Zr) (c1), UiO-66(Zr)-NO_2_ (c2), UiO-66(Zr)-NH_2_ (c3) and MIL-125(Ti)-NH_2_ (**d**).

The selected MOFs under study include the series of Zr-based MOF with UiO-66 topology, namely, UiO-66(Zr), UiO-66(Zr)-NH_2_ and UiO-66(Zr)-NO_2_. It should be remembered that the UiO-66 solids are considered as benchmark Zr-MOF photocatalysts [51]. One of the important features of these MOFs is that the presence of the electron donor or acceptor functional groups in the organic ligand can tune the energy level of the solids [51]. The photocatalytic activity of the most active UiO-66 solid under study was compared with that of MIL-125(Ti)-NH_2_ as reference Ti-MOF [52]. These four MOFs were prepared by solvothermal method as previously reported and described in the SI [49,50]. Figure 2 shows the characteristic diffraction patterns of the four MOFs under study that are in good agreement with their respective simulated patterns and the thermogravimetric analyses of the MOFs that confirmed the thermal stability up to about 350 °C.

**Figure 3:**
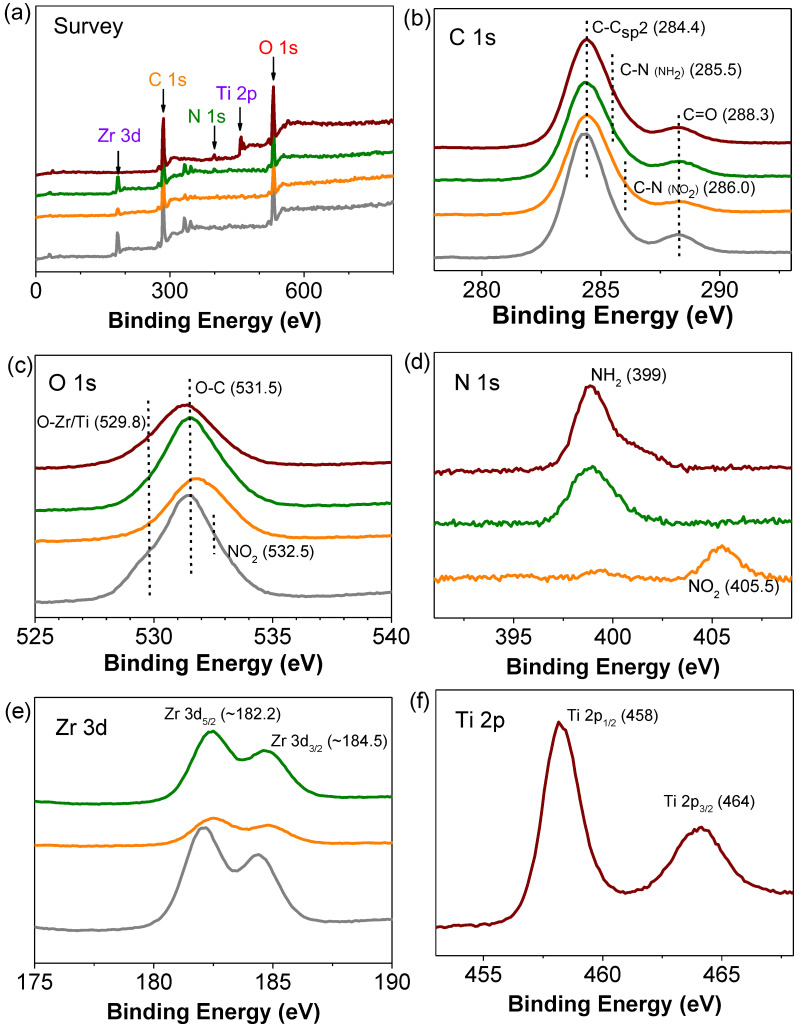
XPS survey spectrum (**a**) and high-resolution C 1s (**b**), O 1s (**c**), N 1s (**d**), Zr 3d (**e**) and Ti 2p (**f**) spectra of the MOFs under study. Legend: UiO-66(Zr) (gray), UiO-66(Zr)-NO_2_ (orange), UiO-66(Zr)-NH_2_ (green) and MIL-125(Ti)-NH_2_ (wine red).

XPS spectroscopy was used to characterize the oxidation state of the elements present in the MOFs under study. Figure 3 compiles the survey and XPS spectra of MOFs, while the corresponding deconvolutions of the elements can be found in the Supplementary Materials (Figures S1–S4). The C 1s spectra show the presence of a band centered at 284.4 eV assigned to C-C sp^2^ of the terephthalate organic ligand. The C 1s spectra of UiO-66(Zr)-NH_2_ and MIL-125(Ti)-NH_2_ exhibit an additional band at 285.5 eV due to the presence of the C-N bond in the amino functional groups. In the case of the UiO-66(Zr)-NO_2_, a band appearing at 286 eV due to the presence of the nitro group can be observed. The O 1s spectra of the four MOFs show a broad band that corresponds to the oxygen atoms present in the carboxylate (about 532 eV) and zirconium or titanium oxo clusters (529.8 eV). In the case of the UiO-66(Zr)-NO_2_ sample, a band due to the oxygen atoms present in the nitro group (~532.5 eV) can be also assigned. The N 1s spectra of the amino- or nitro-based MOFs show their characteristic band centered at about 399 or 405 eV, respectively. The Zr 3d spectra of the UiO-66 samples exhibit two main bands characteristic of the Zr(IV) ions in the oxo clusters at 182.1 and 184.4 eV that correspond to the Zr 3d_5/2_ and Zr 3d_3/2_, respectively. In the case of the MIL-125(Ti)-NH_2_ solid, the XPS Ti 2p due to the presence of Ti(IV) in the octameric oxo cluster resulted in the observation of Ti 2p_1/2_ (458 eV) and Ti 2p_3/2_ (464 eV) signals. In addition, FT-IR spectroscopy confirms the presence of the expected vibration bands of the carboxylate (around 1500 and 1300 cm^−1^), amino (3518–3382 cm^−1^) or nitro (1542 and 1498 cm^−1^) groups present in the terephthalate organic ligand of the MOFs (Figure S5).

The porosity of the MOFs under study was measured by isothermal N_2_ adsorption experiments (Table 1 and Figures S6–S9). The estimated BET surface area and pore volumes are summarized in Table 1. Remarkably is the higher porosity of MIL-125(Ti)-NH_2_ with respect to the UiO-66 solids. SEM measurements showed that all the four MOFs under study exhibit average particle size distribution with values lower than 400 nm (Table 1 and Figures S10 and S11). SEM coupled to EDX reveals a good distribution of the elements in the samples (Figures S12–S15).

**Figure 4:**
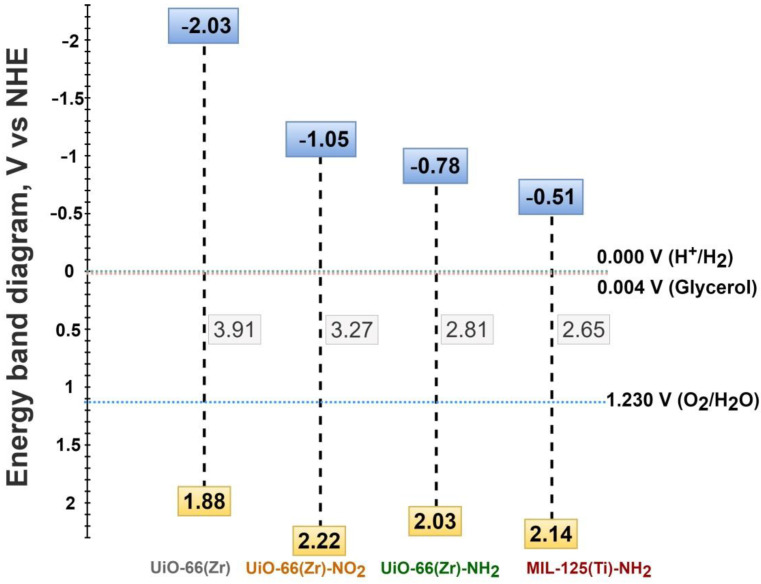
Energy band diagram for UiO-66(Zr)-H, UiO-66(Zr)-NO_2_, UiO-66(Zr)-NH_2_ and MIL-125(Ti)-NH_2_. The proton and oxygen reduction and glycerol oxidation potentials are also indicated.

One of the important aspects in heterogeneous photocatalysis is to determine the energy level of the solid employed for this purpose. Three main factors that determine this diagram in the case of MOFs include their band gap, the highest occupied crystal orbital (HOCO) and the lowest unoccupied crystal orbital (LUCO). Furthermore, the optical properties of the solids were studied by UV-Vis diffuse reflectance spectroscopy. Figure S16 shows that the UiO-66(Zr) exhibits a main broad absorption in the UV range due to the presence of the zirconium oxo clusters and the terephthalate organic ligands. The presence of the nitro and specially the amino group in the terephthalate ligand of the UiO-66(Zr) results in bathochromic shift in the absorption toward visible light region. From these optical absorption data and the corresponding Tauc plots, the optical band gap of UiO-66(Zr), UiO-66(Zr)-NO_2_ and UiO-66(Zr)-NH_2_ was estimated to be 3.91, 3.27 and 2.81 eV, respectively (Figure S16). Similarly, the optical band gap of the MIL-125(Ti)-NH_2_ was estimated as 2.65 eV (Figure S16). These results are in agreement with previous analogous reports and confirm the relatively simplicity to tune the band gap of MOFs by introducing one functional in the organic ligand [53]. Furthermore, XPS spectroscopy was also employed to characterize the valence band maximum of the UiO-66(Zr)-X (X:H, NH_2_, NO_2_) and MIL-125(Ti)-NH_2_ samples (Figure S17). From these values and the optical band gap the conduction band energy minimum was calculated (see details in Supplementary Materials, Section S2. Characterization of the MOF-based materials). Figure 4 illustrates the energy band diagram of the MOF samples under study. The obtained results indicate that the presence of functional groups in the organic ligand reduces the band gap of the solids with respect to the parent MOF and determines the HOCO and LUCO values of the samples. As it is shown later, these energy level diagrams influence to a large extent the observed photocatalytic activity.

### 3.2. Photocatalytic Results

**Figure 5:**
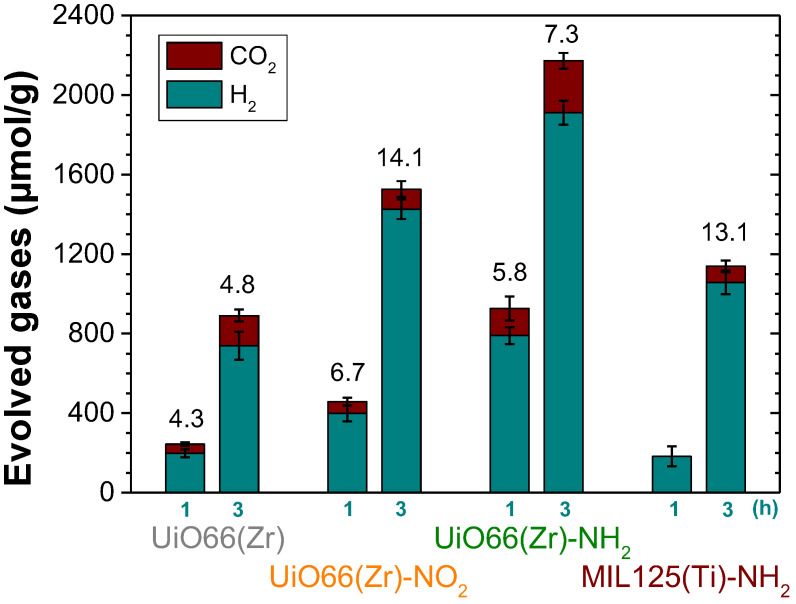
Photocatalytic H_2_ and CO_2_ generation from glycerol aqueous solution using the MOFs under study. The numbers indicated above the bars correspond to the H_2_-to-CO_2_ molar ratio. Reaction conditions: Photocatalyst (5 mg), H_2_O:glycerol mixture (20 mL; 14 vol%), reaction time as indicated, simulated sunlight irradiation (230 W/cm^2^), 35 °C.

Initially, the activity of UiO-66(Zr), UiO-66(Zr)-NH_2_, UiO-66(Zr)-NO_2_ and MIL-125(Ti)-NH_2_ was evaluated for the generation of H_2_ from glycerol photoreforming in aqueous solution under simulated sunlight irradiation. The main role of glycerol is to act as an electron donor during the photocataytic reaction by scavenging the photogenerated holes [45]. During this process, glycerol becomes oxidized through several possible steps, resulting in the formation of CO_2_ and H_2_ (Figure 1). Furthermore, the photogenerated electrons have the ability to reduce protons to H_2_ [45]. Therefore, the evolved hydrogen during the photocatalytic process is determined by the ability of the MOF-based photocatalyst for both glycerol oxidation and proton reduction toward hydrogen production. As previously commented, glycerol photoreforming is a process that involves the generation of H_2_:CO_2_ with a molar ratio of 2.33 (Figure 1a). The results shown in Figure 5 indicate that the UiO-66(Zr)-NH_2_ solid is the most active photocatalyst of the series for the HER with a H_2_:CO_2_ molar ratio of 7.3 after 3 h of reaction. It should be commented that, in all cases, the conversion of glycerol was below 0.5%, and during the photocatalytic reaction, traces of methane were also detected. Furthermore, the used MOF-based photocatalysts retain their crystallinity and morphology, as revealed by XRD and SEM measurements, respectively (Figures S18 and S19). The higher activity of UiO-66(Zr)-NH_2_ with respect to UiO-66(Zr)-NO_2_ or UiO-66(Zr) is attributed to the higher ability of the former to absorb visible light. The higher photoactivity of UiO-66(Zr)-NH_2_ compared to MIL-125(Ti)-NH_2_ is remarkable, even given the slightly higher band gap and lower BET surface area and pore volume of the former solid (Table 1). This observation can be mainly attributed to the more negative LUCO value of UiO-66(Zr)-NH_2_ with respect to MIL-125(Ti)-NH_2_ to favor the HER. In order to put in context of the achieved photocatalytic results, the most active UiO-66(Zr)-NH_2_ was compared with the existing analogous study using MOFs. It should be mentioned that the photocatalytic activity can depend to a large extent on the reaction conditions such as glycerol aqueous solution concentration, catalyst amount, temperature, reactor design or irradiation source and, thus, comparison with literature results should be cautiously taken. With these comments in mind, the achieved activity of UiO-66(Zr)-NH_2_ (0.62 mmol g^−1^ h^−1^ after 3 h; H_2_:CO_2_ 7.3) is higher than that achieved using the Cu-MOF termed as HKUST-1 (0.1 mmol g^−1^ h^−1^ after 8 h, H_2_:CO_2_ ~0.9), both under simulated sunlight irradiation [48].

**Figure 6:**
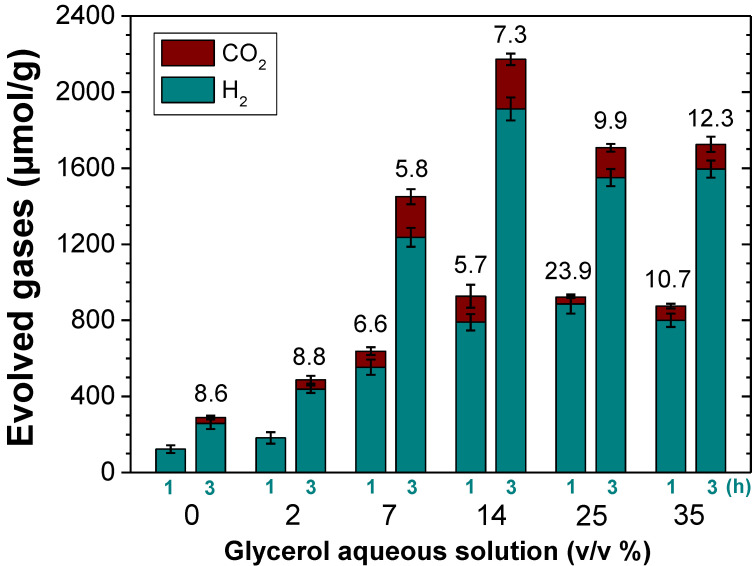
Influence of the glycerol aqueous solution concentration on the photocatalytic H_2_ and CO_2_ generation using UiO-66(Zr)-NH_2_. The numbers indicated above the bars correspond to the H_2_-to-CO_2_ molar ratio. Reaction conditions: Photocatalyst (5 mg), H_2_O:glycerol mixture (20 mL; vol% as indicated), reaction time (as indicated), simulated sunlight irradiation (230 W/cm^2^), 35 °C.

Of particular interest is also the observation of different H_2_-to-CO_2_ ratios as a function of the MOF employed as the photocatalyst. These observations may be associated with the occurrence of different photochemical reactions taking place simultaneously during the photoreforming of glycerol aqueous solutions with each specific MOF (Figure 1). As commented in the introduction, the occurrence of primary or secondary carbon oxidation (carbonyl formation) to a larger extent than the oxidative C-C rupture (Figure 1) would favor a higher H_2_-to-CO_2_ molar ratio. Therefore, it can be assumed that the use of UiO-66(Zr)-NO_2_ as the photocatalyst might favor the carbonyl formation pathway to a larger extent than UiO-66(Zr)-NH_2_ or UiO-66(Zr) and, therefore the higher H_2_-to-CO_2_ molar ratio observed (Figure 5). This fact could be explained by considering the higher oxidation ability of UiO-66(Zr)-NO_2_ with a more positive HOCO value (+2.22 V) than the other two UiO-66 solids (Figure 4). According to previous study, the higher acidity of metals nodes of UiO-66(Zr)-NO_2_ with respect to UiO-66(Zr)-NH_2_ or UiO-66(Zr) may also influence some steps of the reaction mechanism of aqueous glycerol photoreforming [54]. Furthermore, the use of UiO-66 photocatalysts results in an increase in the H_2_-to-CO_2_ molar ratio as the reaction proceeds from 1 to 3 h, and these observations might be associated with the occurrence of the carbonyl pathways to a larger extent at the longer reaction time. For comparison, the similar H_2_-to-CO_2_ ratio at 3 h observed for MIL-125(Ti)-NH_2_ with respect to UiO-66(Zr)-NO_2_ may be also due to the fact that both photocatalysts exhibit similar HOCO values that would favor hydrogen evolution through the carbonyl formation pathway. In the case of MIL-125(Ti)-NH_2_, the formation of H_2_ at 1 h is accompanied with only traces of CO_2_, while as the reaction proceeds, more CO_2_ is formed toward the theoretical 2.33 molar ratio. However, further studies are required to gain more insights about the complex reaction mechanism for glycerol photoreforming using MOF-based materials. Using the most active UiO-66(Zr)-NH_2_ photocatalyst under study, the influence of the glycerol aqueous solution concentration on the H_2_ production was studied. Figure 6 shows that the H_2_ production increases along with the glycerol aqueous solution concentration up to 14 vol%. Further increase in the glycerol concentration resulted in slight decrease in activity. The optimum glycerol concentration observed of 14 vol% to maximize H_2_ production can be interpreted considering that the number of photogenerated electron–hole pairs can efficiently react up to this value. The use of higher glycerol concentration might be responsible for deactivation of the MOF by either poisoning the redox active sites or blocking the porosity. For comparison, the use of methanol instead glycerol as the sacrificial agent, with the same number of moles, results in a relatively higher activity for H_2_ production (4.46 mmol g^−1^ at 3 h) with respect to glycerol (1.85 mmol g^−1^ at 3 h). Considering the relatively higher oxidation potential of methanol (0.016 V vs. NHE) with respect to glycerol (0.004 V vs. NHE) [55], it can be assumed that better diffusion of the former through the porous structure of the UiO-66(Zr)-NH_2_ plays a significant role in the observed photocatalytic activity. Regardless of these comments, it should be remembered that, currently, methanol production is mainly through a catalytic reaction that involves the hydrogenation of CO with H_2_ obtained from steam reforming of hydrocarbons, and, therefore, its use as the sacrificial electron donor for H_2_ production is far from practical applications [6,9]. Therefore, the use of highly available glycerol as a byproduct of biodiesel can contribute simultaneously to the development of this market and to the development of sustainable technologies for H_2_ generation.

**Figure 7:**
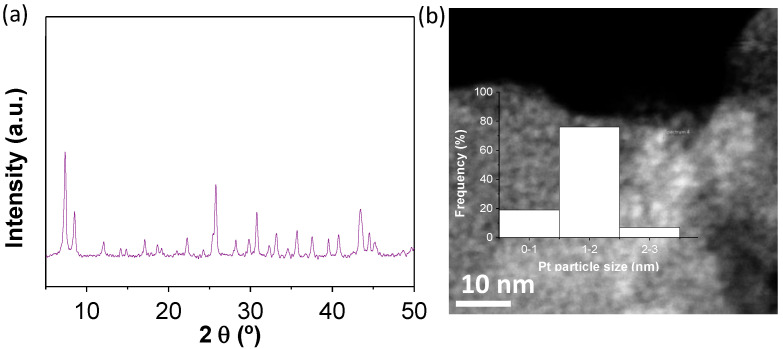
XRD (**a**) and representative DF-STEM image and platinum particle size distribution (**b**) of fresh Pt@UiO-66(Zr)-NH_2_.

To further study the H_2_ generation from the photoreforming of glycerol aqueous solutions, the active UiO-66(Zr)-NH_2_ material was modified with Pt NPs as the co-catalyst. Pt NPs are among the preferred reference noble metal NPs to promote the photocatalytic hydrogen generation [47]. For example, previous studies have reported the use of Pt NPs supported on MOFs such as UiO-66(Zr)-NH_2_ as photocatalysts for the HER using methanol or TEOA as sacrificial agents [56,57,58]. Frequently, the use of Pt NPs loadings below 3 wt% is considered an adequate strategy to boost the photocatalytic activity for HER reaction [56,58]. In the present study, the innovation resides in the use for the first time of Pt NPs-supported UiO-66(Zr)-NH_2_ at low metal loading (1 wt%) as photocatalyst for hydrogen production in a sustainable manner from glycerol photoreforming. PXRD of the as-prepared Pt NPs-supported UiO-66(Zr)-NH_2_ by the photodeposition method shows that the resulting MOF-based material retains its initial crystallinity (Figure 7a). DF-STEM of the Pt@UiO-66(Zr)-NH_2_ reveals an average particle size and standard deviation of Pt NPs of 1.27 and 0.43 nm, respectively (Figure 7b). Point EDX analyses confirmed the presence of Pt NPs within the MOF framework (Figure S20).

**Figure 8:**
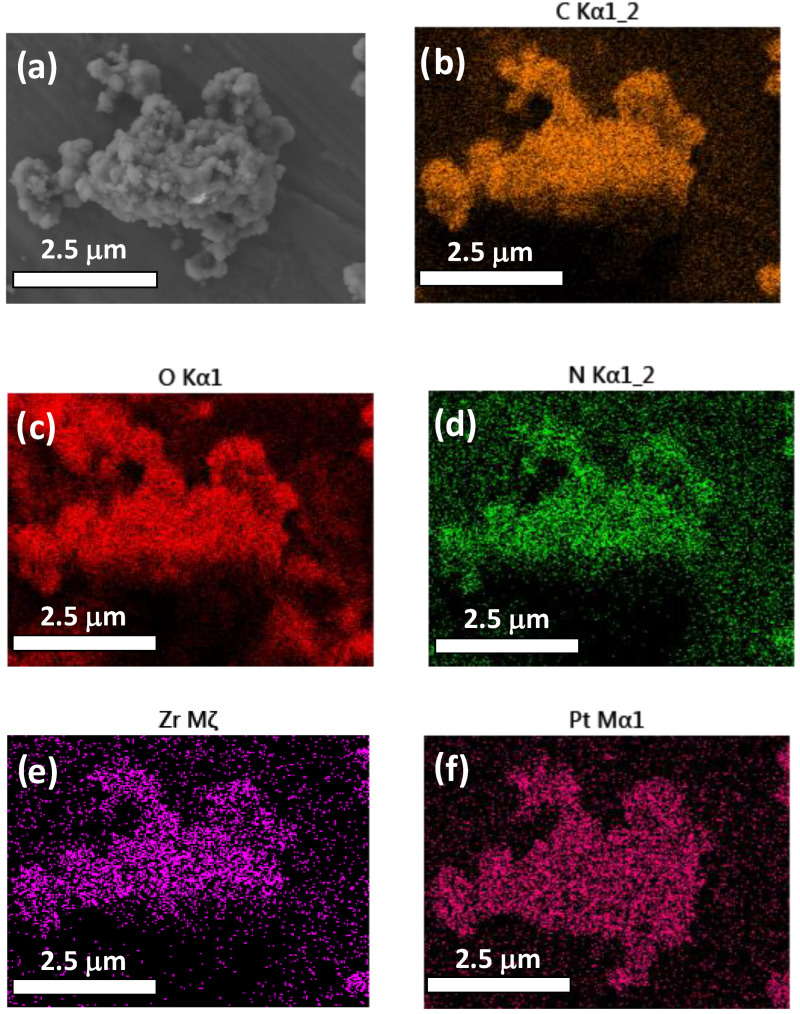
HR-SEM image of Pt@UiO-66(Zr)-NH_2_ (**a**) and EDX mapping: carbon (**b**), oxygen (**c**), nitrogen (**d**), zirconium (**e**), platinum (**f**).

SEM measurements coupled to EDX analysis support that the small Pt NPs are well-distributed through the UiO-66(Zr)-NH_2_ network (Figure 8). XPS allows for confirmation of the presence of supported Pt NPs in UiO-66(Zr)-NH_2_ in its metallic form (Figure S21) based on the binding energies of Pt 4f_7/2_ and 4f_5/2_ at 69.6 and 72.6 eV, respectively.

**Figure 9:**
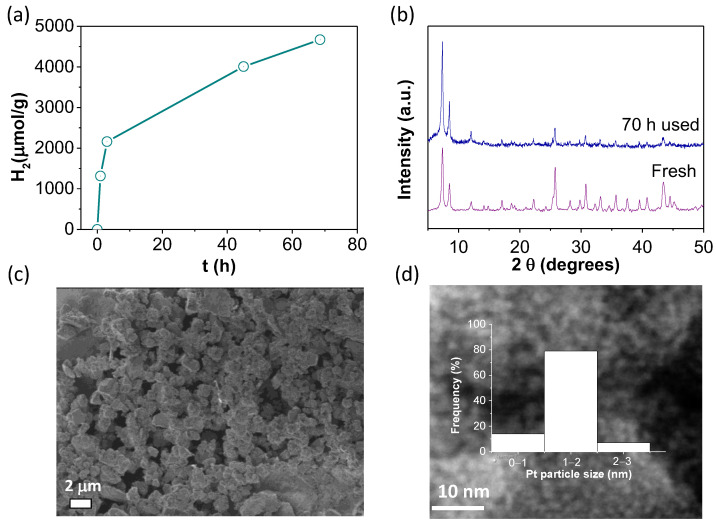
(**a**) H_2_ evolution reaction after 70 h. (**b**) XRD patterns to confirm the stability of Pt@UiO-66(Zr)-NH_2_. (**c**) Representative SEM and DF-STEM images of used Pt@UiO-66(Zr)-NH_2_ together with platinum particle size distribution (**d**). Reaction conditions: Photocatalyst (5 mg), H_2_O:glycerol mixture (20 mL; 14 vol%), reaction time as indicated, simulated sunlight irradiation (230 W/cm^2^), 35 °C.

After the characterization of the Pt@UiO-66(Zr)-NH_2_ sample, this solid was employed as the photocatalyst for H_2_ generation from glycerol aqueous solution (14 vol%). The results show higher H_2_ photocatalytic production when using Pt@UiO-66(Zr)-NH_2_ (2.3 mmol g^−1^ in 3 h) with respect to the parent UiO-66(Zr)-NH_2_ (1.7 mmol g^−1^ in 3 h). Remarkably, the Pt@UiO-66(Zr)-NH_2_ material can be used under the same reaction conditions for 70 h (Figure 9a) while retaining its crystallinity and morphology, as revealed by XRD and SEM measurements, respectively (Figure 9b,c). DF-STEM measurements (Figure 9d) coupled with EDX analyses (Figure S22) also allowed us to confirm the absence of significant Pt NP aggregation (1.34 ± 0.45 nm) with respect to the fresh sample (1.27 ± 0.43 nm). 

### 3.3. Reaction Mechanism

**Figure 10:**
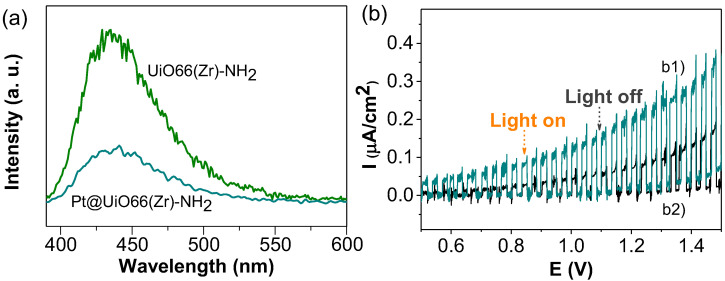
(**a**) PL of UiO-66(Zr)-NH_2_ and Pt@UiO-66(Zr)-NH_2_ upon excitation at 340 nm. (**b**) Photocurrent measurements upon electrode polarization and illumination/dark cycles using Pt@UiO-66(Zr)-NH_2_/FTO as the working electrode in the presence (b1) and absence (b2) of glycerol.

To gain some understanding about the photocatalytic performance and reaction mechanism of Pt@UiO-66(Zr)-NH_2_ and UiO-66(Zr)-NH_2_ solids, several spectroscopic and electrochemical measurements were carried out. Initially, the PL response of Ar-purged acetonitrile MOF suspensions having the same absorbance value (ca 0.35) at 340 nm, corresponding with the excitation wavelength of the organic ligand, was measured. Figure 10 shows that the presence of Pt NPs (1 wt%) within the UiO-66(Zr)-NH_2_ solid results in a decrease in PL of 66% with respect to the parent UiO-66(Zr)-NH_2_. These results are interpreted in a way that considers the fact that the presence of Pt NPs avoids, at least partially, the photoinduced charge recombination that is the process responsible for the observed photoluminescence emission. In fact, photocurrent measurements using a Pt@UiO-66(Zr)-NH_2_/FTO (fluorine doped tin oxide) working electrode upon polarization and dark/illumination cycles allow us to confirm the occurrence of photoinduced charge separation, a process that is favored in the presence of glycerol. This enhancement of photocurrent associated with the role of glycerol as the sacrificial electron donor can be explained considering that it quenches the photogenerated holes and increases the photocurrent intensity. This effect agrees with the previous photocatalytic data in which the presence of glycerol in water increases the photocatalytic H_2_ production (Figure 6). 

**Figure 11:**
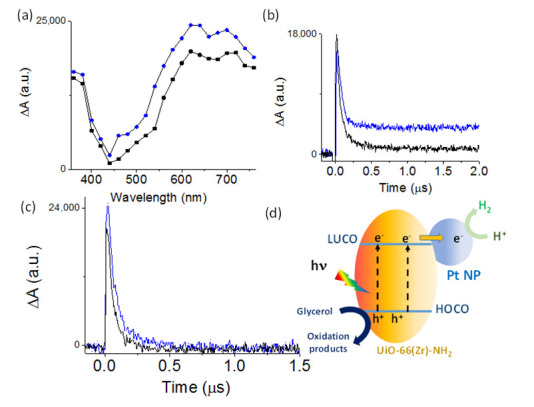
(**a**) Transient absorption spectra for UiO-66(Zr)-NH_2_ (●) and Pt@UiO-66-NH_2_ (■) upon excitation at 365 nm under Ar atmosphere recorded at 25 ns. Transient absorption decay kinetics for UiO-66(Zr)-NH_2_ (blue line) and Pt@UiO-66(Zr)-NH_2_ (black line) recorded at 380 (**b**) or 680 (**c**) nm under Ar. (**d**) Simplified illustration of the proposed reaction mechanism using Pt@UiO-66(Zr)-NH_2_.

TAS was also used to further study the reaction mechanism when using UiO-66(Zr)-NH_2_ or Pt@UiO-66(Zr)-NH_2_ as photocatalysts. Specifically, TAS spectrum of both MOFs recorded upon irradiation at ligand-centered absorption at 355 nm under Ar atmosphere clearly shows the presence of two bands (Figure 11a). The first band appearing from 350 to 450 nm and second one from 550 to 750 nm, both exhibiting different decays of kinetics and profiles. This fact suggests that these two absorbance bands correspond to different species. In the case of UiO-66(Zr)-NH_2_, the first region is characterized by transient absorbance decay kinetics with two components, one quick and intense, attributed to the occurrence of charge separation, and other residual, with a longer lifetime due to charge delocalization along the MOF crystal (Figure 11b). The second band observed in the region from 550–750 nm is mainly characterized by one component decay (Figure 11c, blue line). In the case of the Pt@UiO-66(Zr)-NH_2_ sample, the presence of Pt mainly causes the quenching of the second component, inhibiting photoinduced charge delocalization in the MOF (Figure 11b). More specifically, the transient absorbance lifetime decay of UiO-66(Zr)-NH_2_ (65 ns) is quenched in the presence of Pt NPs in UiO-66(Zr)-NH_2_ (40 ns) (Figure 11c). In addition, the presence of Pt NPs results in a quenching of the transient absorbance spectrum of UiO-66(Zr)-NH_2_, particularly intense in the region from 550 to 750 nm. These observations can be interpreted considering that metallic Pt NPs have the ability of trapping photoinduced generated charges. 

Aiming to bring light to the nature of the observed photoinduced charge separation species for the most active Pt@UiO-66(Zr)-NH_2_ sample, additional TAS measurements in the presence of quenchers were carried out. Methanol was selected as the hole quencher due to its good electron-donor behavior, while molecular oxygen was chosen as the electron acceptor. Figures S23 and S24 show that the use of these two quenchers results in significant changes to the TAS measurements. In particular, the presence of methanol quenches the intensity signal and the spectra decay along the whole spectrum, specifically the region between 350 and 450 nm (Figure S23). These observations suggest that the transient absorption observed mainly from 350 to 450 nm corresponds to photogenerated holes. The use of O_2_ as the quencher also modifies the transient absorption profiles and decreases the decay lifetimes (Figure S24). For instance, the presence of O_2_ decreases the decay lifetimes from 65 to 38 ns recorded at 380 nm or from 45 to 30 ns at 680 nm. The observed quenching of transient absorbance intensity due to the presence of O_2_ in the region from 550 to 750 nm is associated with the presence of photogenerated electrons. These observations agree with the role of supported Pt NPs as an electron reservoir responsible for the observed decrease in both signal intensity and lifetime of the transient species in the region from 550 to 750 nm with respect to the parent MOF. Figure 11d illustrates a simplified reaction mechanism showing the photoinduced electron transfer from the organic ligand to the metal node of the MOF. Then, the electrons present in the LUCO are transferred to Pt NPs where the reduction reactions occur, while oxidations take place in the HOCO localized in the organic ligand.

**Figure 12:**
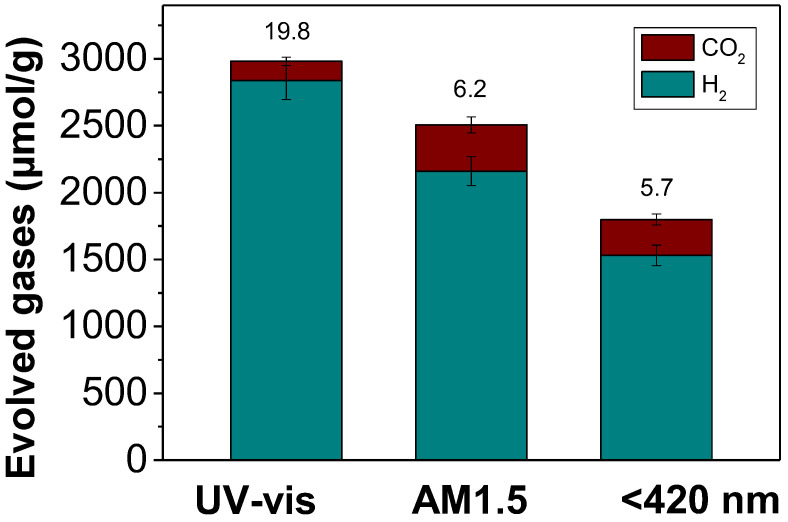
Evolved gases over the influence of the light irradiation source with Pt@UiO-66(Zr)-NH_2_. Reaction conditions: Photocatalyst (5 mg), H_2_O:glycerol mixture (20 mL; 14 vol%), reaction time 3 h, irradiation source as indicated, 35 °C.

In addition to the above spectroscopic and electrochemical characterization, the influence of the light irradiation source with Pt@UiO-66(Zr)-NH_2_ was also investigated. Figure 12 shows the highest photocatalytic activity achieved using UV-Vis light, followed by simulated sunlight irradiation and then visible light irradiation. In any case, these results confirm unambiguously that Pt@UiO-66(Zr)-NH_2_ behaves as an efficient photocatalyst under both visible or simulated sunlight irradiation. 

## 4. Conclusions

The present study has shown the possibility of using UiO-66 topology for H_2_ generation from photoreforming of glycerol aqueous solutions under simulated sunlight irradiation. The most active photocatalyst of the series was UiO-66(Zr)-NH_2_, followed by UiO-66(Zr)-NO_2_ and the less active UiO-66(Zr). The activity of MIL-125(Ti)-NH_2_ was lower than that of UiO-66(Zr)-NH_2_. The observed order of activity was mainly attributed to the energy level difference of the solids, where UiO-66(Zr)-NH_2_ has an appropriate band gap to absorb visible light together with adequate LUCO value to promote reduction reactions. Deposition of Pt NPs as the co-catalyst for hydrogen generation within the UiO-66(Zr)-NH_2_ network has significantly improved the photocatalytic activity of the MOF. In addition, the Pt@UiO-66(Zr)-NH_2_ photocatalyst was employed for about 70 h with good H_2_ generation and without loss of crystallinity or change in its morphology based on PXRD and SEM measurements, respectively. Furthermore, Pt particle size distribution was also retained, as revealed by TEM measurements. Evidence in support of the occurrence of photoinduced charge separation with Pt@UiO-66(Zr)-NH_2_ was obtained by TAS and photocurrent measurements. In addition, PL measurements using Pt@UiO-66(Zr)-NH_2_ and UiO-66(Zr)-NH_2_ indicated that the use of the Pt NPs avoids to some extent the occurrence of undesirable photoinduced charge recombination. The study of the influence of light irradiation on H_2_ generation from glycerol aqueous solution using Pt@UiO-66(Zr)-NH_2_ revealed that this photocatalyst exhibits good activity under both visible and simulated sunlight irradiation. The authors consider that this study will contribute to the field of solar-driven photocatalytic H_2_ generation using MOFs and sustainable feedstocks as sacrificial agents.

## Figures and Tables

**Table 1:** BET surface area, pore volume and average particle size and standard deviation of the MOFs under study.

	BET Surface Area (m^2^/g)	Pore Volume (cm^3^/g)	Average Particle Size and Standard Deviation (nm) ^a^
UiO-66(Zr)	650	0.23	114/92
UiO-66(Zr)-NO_2_	782	0.42	109/48
UiO-66(Zr)-NH_2_	922	0.43	366/201
MIL-125(Ti)-NH_2_	1046	0.51	146/60

^a^ Obtained from SEM measurements.

## Data Availability

The data presented in this study are available on request from the corresponding authors.

## Supplementary Materials

The following supporting information can be downloaded at: https://www.mdpi.com/article/10.3390/nano12213808/s1, Figure S1: XPS of UiO-66(Zr): survey (a), C 1s (b), O 1s (c), Zr 3d (d), Figure S2: XPS of UiO-66(Zr)-NO_2_: survey (a), C 1s (b), O 1s (c), N 1s (d), Zr 3d (e), Figure S3: XPS of UiO-66(Zr)-NH_2_: survey (a), C 1s (b), O 1s (c), N 1s (d), Zr 3d (e), Figure S4: XPS of MIL-125(Ti)-NH_2_: survey (a), C 1s (b), O 1s (c), N 1s (d), Ti 2p (e), Figure S5: FT-IR spectroscopy of UiO-66(Zr) (a), UiO-66(Zr)-NO_2_ (b), UiO-66(Zr)-NH_2_ (c) and MIL-125(Ti)-NH_2_ (d), Figure S6: Isothermal N_2_ adsorption curve of UiO-66(Zr), Figure S7: Isothermal N_2_ adsorption curve of UiO-66(Zr)-NO_2_, Figure S8: Isothermal N_2_ adsorption curve of UiO-66(Zr)-NH_2_, Figure S9: Isothermal N_2_ adsorption curve of MIL-125(Ti)-NH_2_, Figure S10: HR-SEM of (a) UiO-66(Zr)-H, (b) UiO-66(Zr)-NO_2_, (c) UiO-66(Zr)-NH_2_ and (d) MIL-125(Ti)-NH_2_, Figure S11: Particle size distribution of (a) UiO-66(Zr)-H, (b) UiO-66(Zr)-NO_2_, (c) UiO-66(Zr)-NH_2_ and (d) MIL-125(Ti)-NH_2_, Figure S12: HR-SEM image of UiO66-(Zr) (a) and EDX mapping: carbon (b), oxygen (c), zirconium (d), Figure S13: HR-SEM image of UiO66-(Zr)-NO_2_ (a) and EDX mapping: carbon (b), oxygen (c), nitrogen (d), zirconium (e); Figure S14: HR-SEM image of UiO66-(Zr)-NH_2_ (a) and EDX mapping: carbon (b), oxygen (c), nitrogen (d), zirconium (e), Figure S15: HR-SEM image of MIL-125-(Ti)-NH_2_ (a) and EDX mapping: carbon (b), oxygen (c), nitrogen (d), titanium (e), Figure S16: UV-Vis diffuse reflectance spectroscopy and its corresponding tauc plot: (a) UiO-66(Zr)-H, (b) UiO-66(Zr)-NO_2_, (c) UiO-66(Zr)-NH_2_ and (d) MIL-125(Ti)-NH_2_, Figure S17: Valence band of (a) UiO-66(Zr)-H, (b) UiO-66(Zr)-NO_2_, (c) UiO-66(Zr)-NH_2_ and (d) MIL-125(Ti)-NH_2_, Figure S18: (a) XRD patterns of used UiO-66(Zr) (a1), UiO-66(Zr)-NO_2_ (a2) and UiO-66(Zr)-NH_2_ (a3). (b) XRD patterns of used MIL-125(Ti)-NH_2_, Figure S19: HR-SEM of used (a) UiO-66(Zr)-H, (b) UiO-66(Zr)-NO_2_, (c) UiO-66(Zr)-NH_2_ and (d) MIL-125(Ti)-NH_2_, Figure S20: Representative DF-STEM image (a) and point EDX analysis of fresh Pt/UiO-66(Zr)-NH_2_ (b), Figure S21: (a) XPS C 1s (b), O 1s (c), N 1s (d), Zr 3d (e) and Pt 4f (f) of 1%wtPt@UiO-66(Zr)-NH_2_, Figure S22: Representative DF-STEM image (a) and point EDX analysis of the Pt/UiO-66(Zr)-NH_2_ photocatalyst used for 70 h (b), Figure S23: TAS spectra of Pt@UiO-66(Zr)-NH_2_ purged with argon (■) and after adding methanol (●) (a). Absorbance transition decay of Pt@UiO-66(Zr)-NH_2_ before (black line) and after adding methanol (red line) recorded at 380 nm (b) and 680 nm (c), Figure S24: TAS spectra of Pt@UiO-66(Zr)-NH_2_ purged with argon (■) and after adding molecular oxygen (●) (a). Absorbance transition decay of Pt@UiO-66(Zr)-NH_2_ before (black line) and after adding molecular oxygen (red line) recorded at 380 nm (b) and 680 nm (c).
