# Supplementary material for: Photocatalytic Hydrogen Production from Glycerol Aqueous Solutions as Sustainable Feedstocks Using Zr-Based UiO-66 Materials under Simulated Sunlight Irradiation
Source: Nanomaterials (Basel). 2022 Oct 28;12(21):3808. doi: 10.3390/nano12213808 (PMC9658527; doi:10.3390/nano12213808)
Supplement: Supplementary file 1 [file nanomaterials-12-03808-s001.zip › nanomaterials-1985428-supplementary.pdf]

Supplementary Material

Photocatalytic Hydrogen Production from Glycerol Aqueous Solutions as Sustainable Feedstocks Using Zr-Based UiO-66 Materials under Simulated Sunlight Irradiation

Celia M. Rueda-Navarro, Belén Ferrer, Herme G. Baldoví * and Sergio Navalón *

Departamento de Química, Universitat Politècnica de València, C/Camino de Vera s/n, 46022 Valencia, Spain
* Correspondence: hergarba@cam.upv.es (H.G.B.); sernaol@doctor.upv.es (S.N.)

1. Synthesis of the MOF-based materials

Synthesis of UiO-66(Zr). $ZrCl_4$ (0.466 g, 2 mmol) and terephthalic acid (0.332 g, 2 mmol) were mixed with N,N′- dimethylformamide (DMF, 6 mL), the system was sonicated for 20 min (450 W power) and then, transferred to a Teflon-lined autoclave.[1] The autoclaved was sealed and placed in a pre-heated oven at 220 °C and maintaining the temperature for 12 h. After cooling the system to room temperature, the resulting white solid was recovered by filtration, and washed several times with DMF and methanol while stirring. The recovered solid was further washed in a Soxhlet for 4 h using methanol as solvent. The solid was dried under vacuum at 150 °C overnight.

Synthesis of UiO-66(Zr)-NH_2. $ZrCl_4$ (0.466 g, 2 mmol) and 2-amino-terephthalic acid (0.362 g, 2 mmol) were mixed with DMF (6.0 mL), the system sonicated for 20 min and then, the mixture transferred in a Teflon-lined autoclave.[1] The sealed autoclave was placed in a pre-heated oven at 100 ˚C for 24 hours. After cooling the system at room temperature, the solid was recovered by filtration, washed several times with DMF and methanol, washed in a Soxhlet for 4 h using methanol, and dried at room temperature.

Synthesis of UiO-66(Zr)-NO_2. $ZrCl_4$ (0.862 g, 3.699 mmol), nitro-terephthalic acid (0.620 g, 3.701 mmol) and H_2O (0.200 mL, 11.10 mmol) were mixed with DMF (100 mL, 1291 mmol) in 250 ml volumetric flask.[2] The system was heated at 70 °C and stirred during the preparation to ensure the complete dissolution of the reagents. Once all reagents were dissolved, the flask was placed in a pre-heated oven to 120 °C for 72 hours after removing the stirring magnet and closing the system. The resulting precipitate was recovered by centrifugation. Then, it was washed with DMF and methanol several times. It was further washed in a Soxhlet for 4 h using methanol as solvent. And finally, the powder was dried under vacuum at 150 °C overnight.

Synthesis of MIL-125(Ti)-NH_2. 2-aminoterephthalic acid (1.43 g, 7.9 mmol) was initially dissolved in anhydrous N, N-dimethylformamide (DMF, 20 mL) and then, anhydrous methanol (5 mL) was added to the flask. The system was sonicated for 20 min (450 W powder) and then, the reaction mixture transferred to a Teflon-lined autoclave (50 mL) where titanium isopropoxide (1.36 g, 4.8 mmol) was added. The autoclave was then sealed, heated up to 110 °C for 72 h and cooled down to room temperature. The resulting precipitate was recovered by filtration, washed with DMF at room temperature for 12 h under stirring, and then, washed with DMF at 70 °C. This washing procedure was re-peated using methanol as solvent. The recovered solid was further washed in a Soxhlet for 4 h using methanol as solvent. The recovered solid by filtration was dried in an oven at 100 ºC for 24 h.

Deposition of Pt NPs on UiO-66(Zr)-NH_2. Pt NPs were deposited in the as-prepared UiO-66(Zr)-NH_2 material using the so-called photodeposition method.[1] Briefly, the MOF (50 mg) was dispersed in a mixture of Milli-Q water (8 mL) and methanol (13 mL) using a quartz tube. Subsequently, the corresponding amount of

$H_2PtCl_6\cdot(H_2O)_6$ previously dissolved in water (1 mL) was added to this quartz tube. Then, the system was purged with Ar for 30 min and immediately irradiated using a UV-vis light lamp (150 W) for 4 h. The resulting solid was recovered by filtration, washed several times with Milli-Q water and dried in an oven at 100 °C for 24 h.

2. Characterization of the MOF-based materials

Powder X-ray diffraction (PXRD) data were recorded on a Philips XPert diffractometer equipped with a graphite monochromator (40 kV and 45 mA) employing Ni filtered Cu K_α radiation.

Isothermal nitrogen adsorption data was collected using an ASAP 2010 Micromeritics device.

Scanning electron microscopy (SEM) images were collected on a scanning electron microscope (SEM, Zeiss instrument, AURIGA Compact) equipped with an energy-dispersive X-ray (EDX) detector. Scanning transmission electron microscopy images in dark field (DF-STEM) were recorded on a JEOL JEM2100F instrument operating at 200 kW.

Pt nanoparticle size distribution was estimated by measuring more than 200 particles from the Pt@UiO-66(Zr)-NH_2 sample.

X-ray photoelectron spectra (XPS) were recorded using a SPECS spectrometer equipped with an MCD-9 detector and using a monochromatic Al (K_α= 1486.6 eV) X-ray source. Spectra deconvolution was performed using the CASA software using the C 1s peak at 284.4 eV as binding energy reference. XPS valence band maximum versus the Fermi level ($E^f{}_\upsilon$) was estimated from the intersection of the flat XPS energy and the linear fit of the leading valence band edge in the XPS graph. The valence band maximum versus the NHE can be calculated from the equation, $E_\upsilon{}^{NHE} = E_\upsilon{}^f + \phi_{sp}$ -4.44 where ϕ_{sp} is the work function of the spectrometer with the value 4.244 eV. The conduction band minimum can be determined from this value and the optical band gap estimated by the Tauc plot.

Photocurrent measurements were performed using a standard three-electrode electrochemical cell. The working electrons were composed by a transparent fluoride-doped tin oxide (FTO)-coated glass substrate on which a thin layer of the Pt@UiO-66(Zr)-NH_2 or UiO-66(Zr)-NH_2 samples were deposited. A platinum wire was used as counter electrode and a standard calomel electrode (SCE) as the reference electrode. To remove the oxygen present in the cell the system was purged through the electrolyte solution with Ar for 15 min. The photocurrent was measured under dark or illumination conditions under continuous polarization of the working electrode at potentials from 1.5 to -0.5 V. The illumination of the working electrode was performed using an optical fibber connected to a 150 W Hg-Xe lamp.

Laser flash photolysis measurements were performed with an OPO System Ekspla (EKS-NT342C-10) coupled with an UV extension (EKS-NT342C-SH-SFG) as the excitation source and an Edinburgh Instruments detection System (LP980) coupled with an ICCD camera (Andor iStar CCD 320T). The OPO System Ekspla light sources was adjusted to 340 nm. Similar acetonitrile suspensions than those employed for the PL measurements were also used in this case. In some cases, the cuvette was purged for 10 min with O_2 or N_2O as electron quenchers. In other cases, methanol (100 μL) was included to the cuvette as hole quencher and the system again sonicated with Ar for 10 min.

Photoluminescence (PL) measurements were carried out using JASCO FP-8500 instrument. For this purpose, Ar-purged acetonitrile suspensions of Pt@UiO-66(Zr)-NH_2 or UiO-66(Zr)-NH_2 with an adjusted absorption at 0.35 a.u. at the excitation wavelength of 340 nm were prepared using a quartz cuvette.

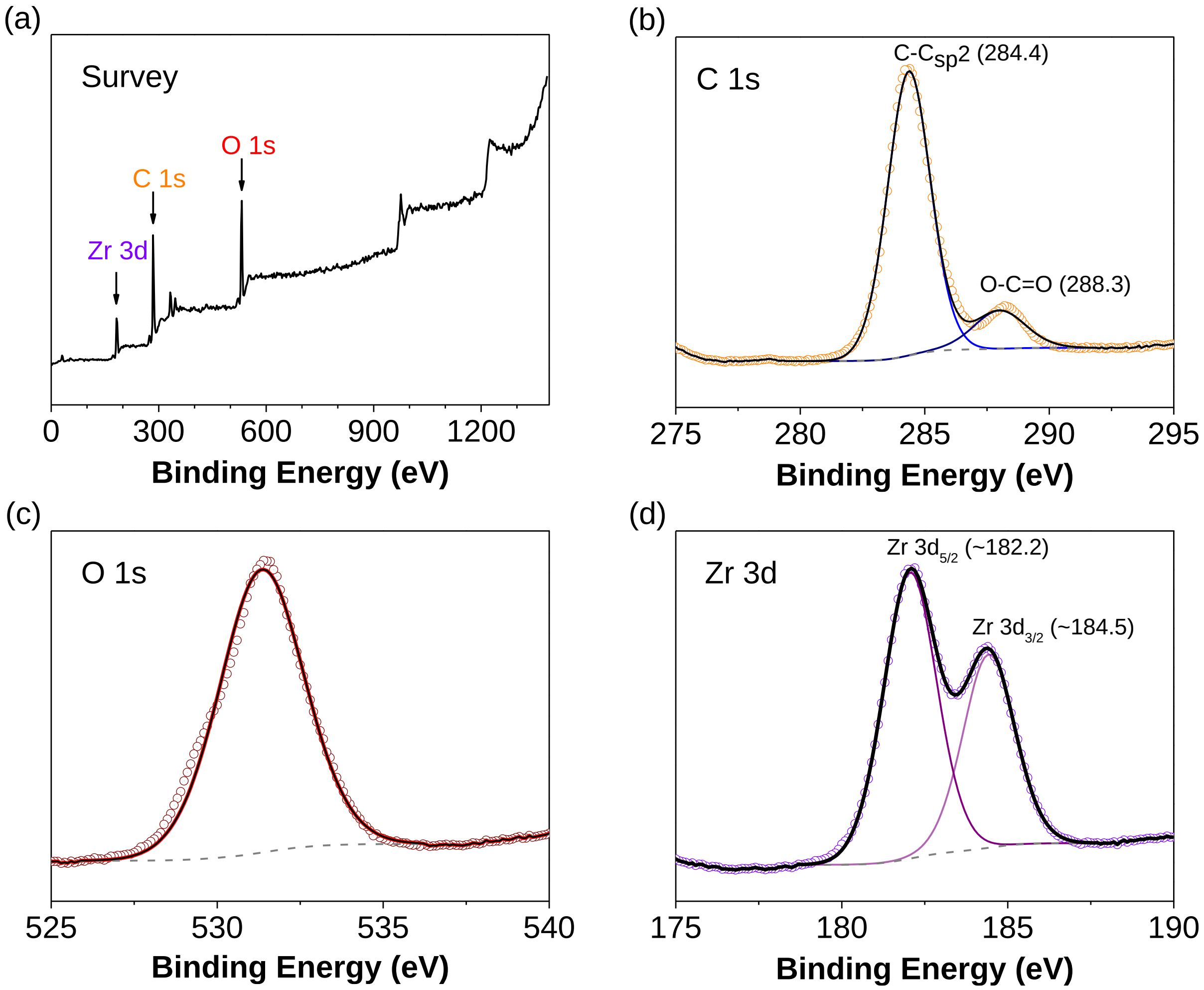

Figure S1. XPS of UiO-66(Zr): survey (**a**), C 1s (**b**), O 1s (**c**), Zr 3d (**d**).

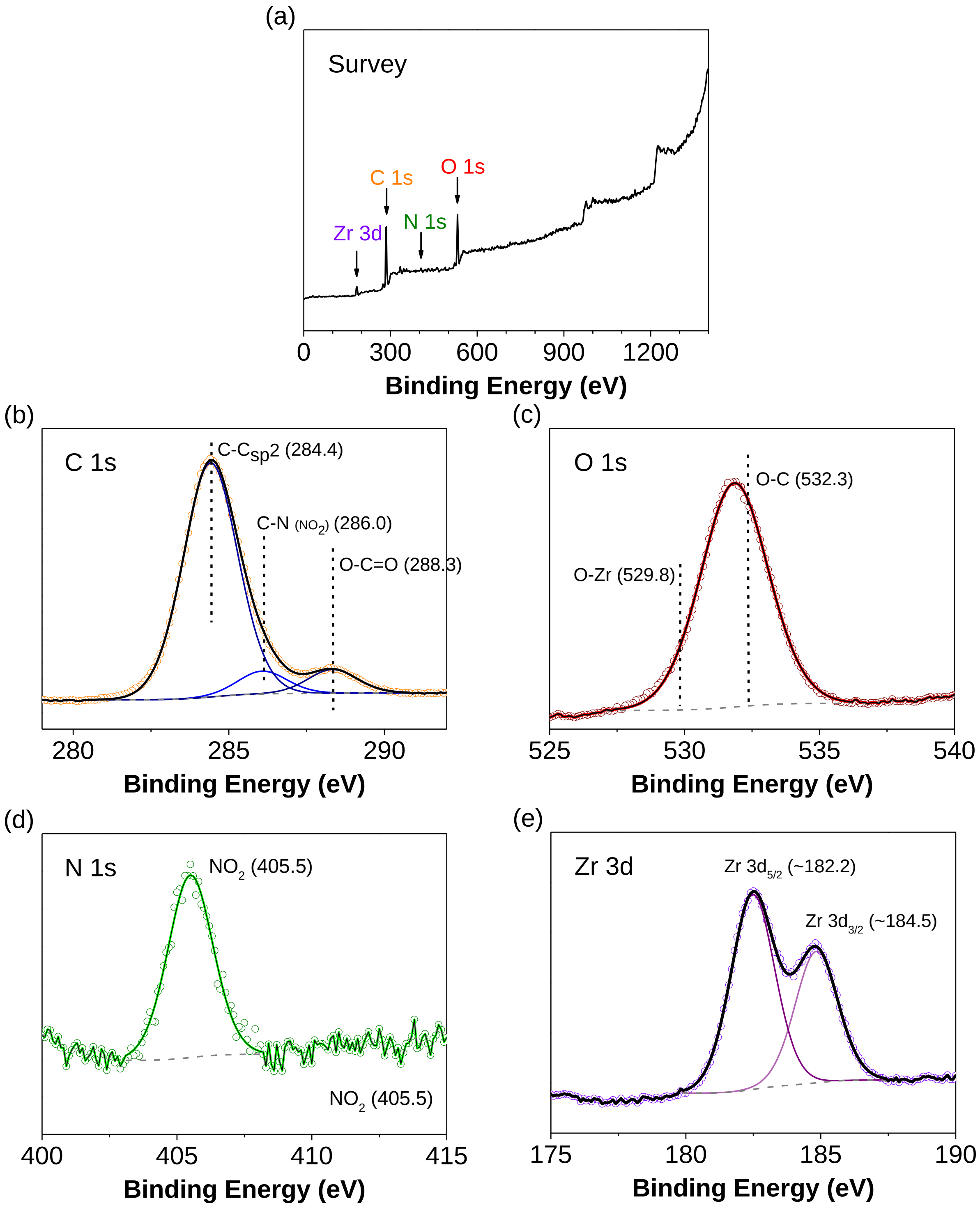

Figure S2. XPS of UiO-66(Zr)-NO_2: survey (**a**), C 1s (**b**), O 1s (**c**), N 1s (**d**), Zr 3d (**e**).

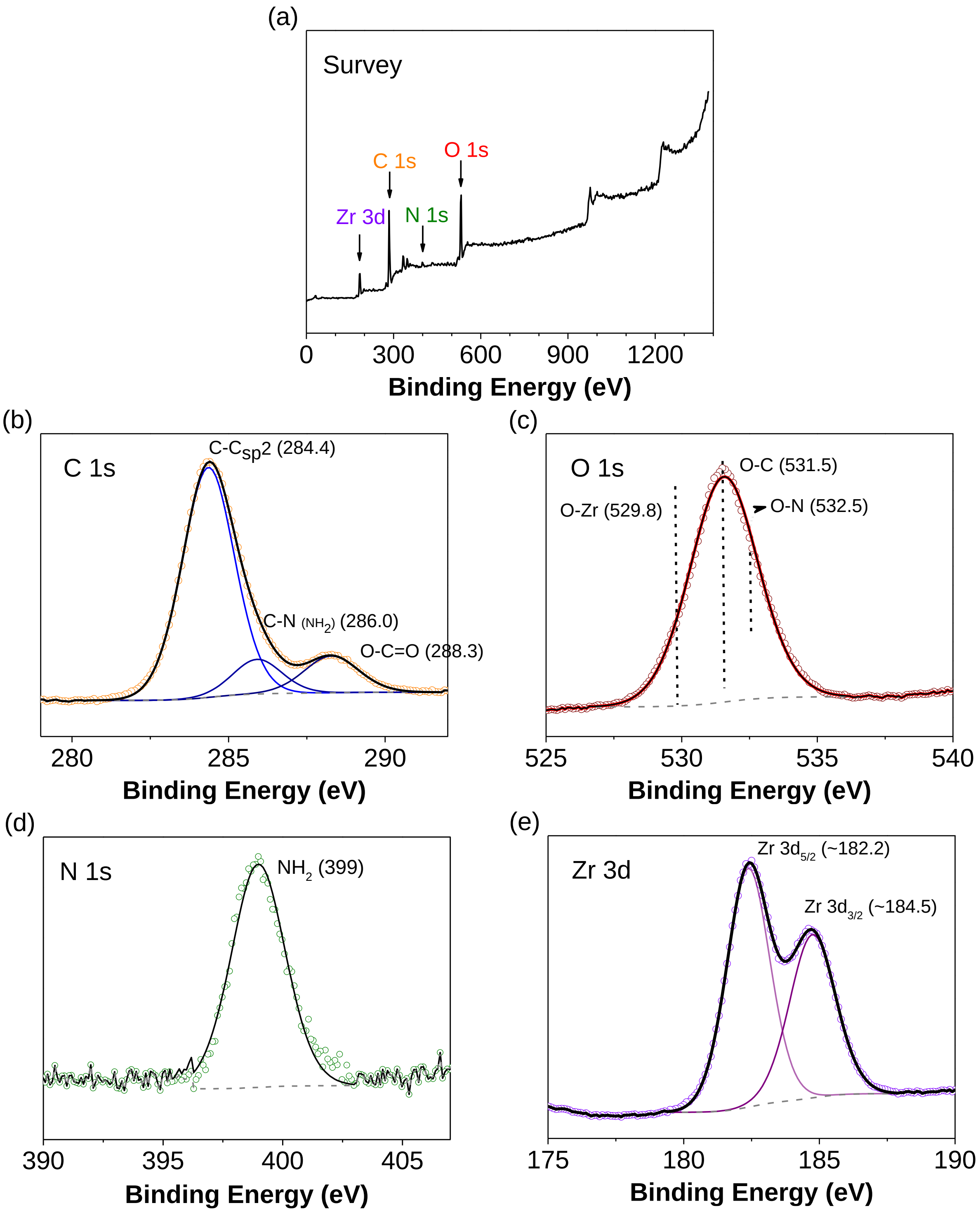

Figure S3. XPS of UiO-66(Zr)-NH2: survey (**a**), C 1s (**b**), O 1s (**c**), N 1s (**d**), Zr 3d (**e**).

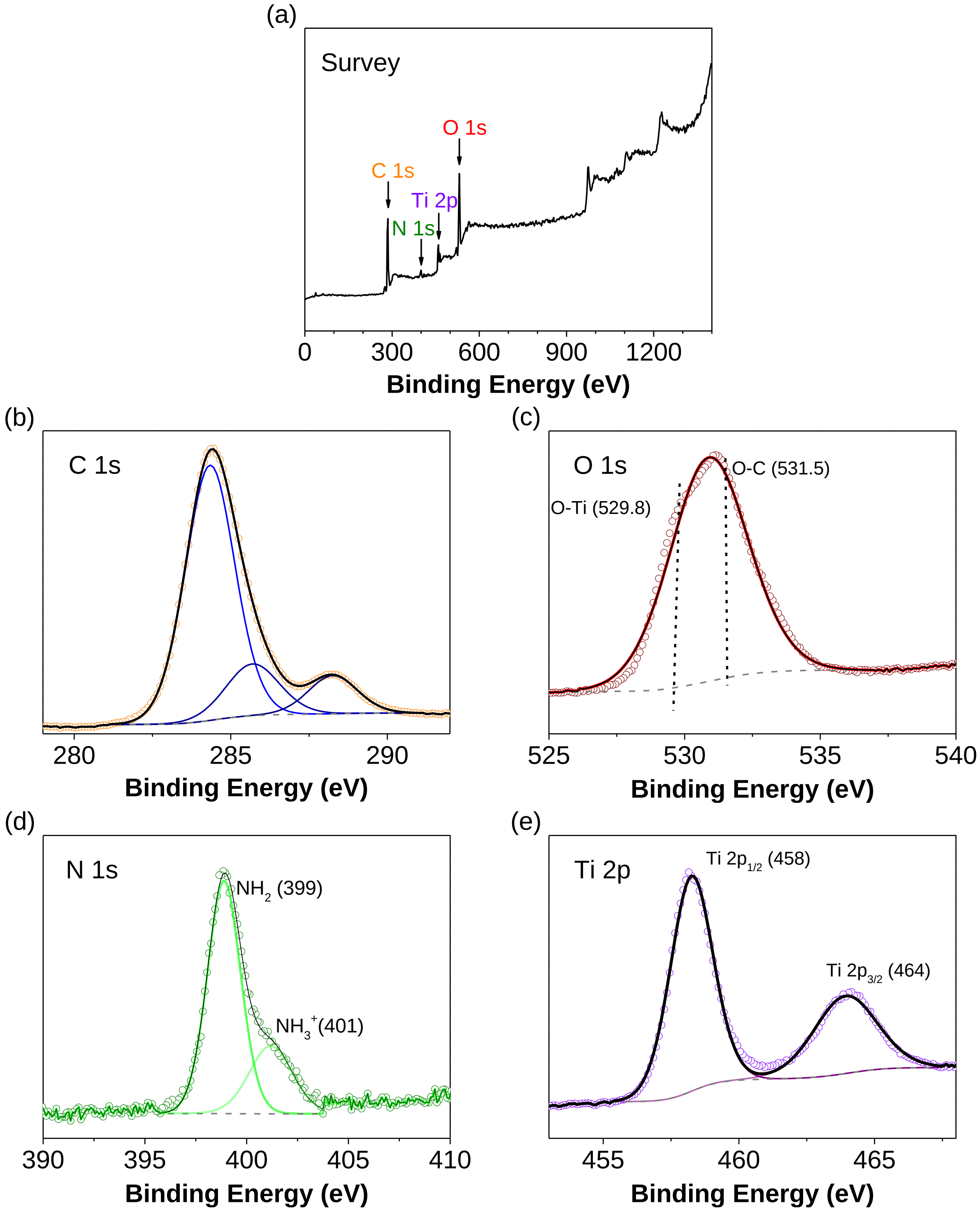

Figure S4. XPS of MIL-125(Ti)-NH_2: survey (**a**), C 1s (**b**), O 1s (**c**), N 1s (**d**), Ti 2p (**e**).

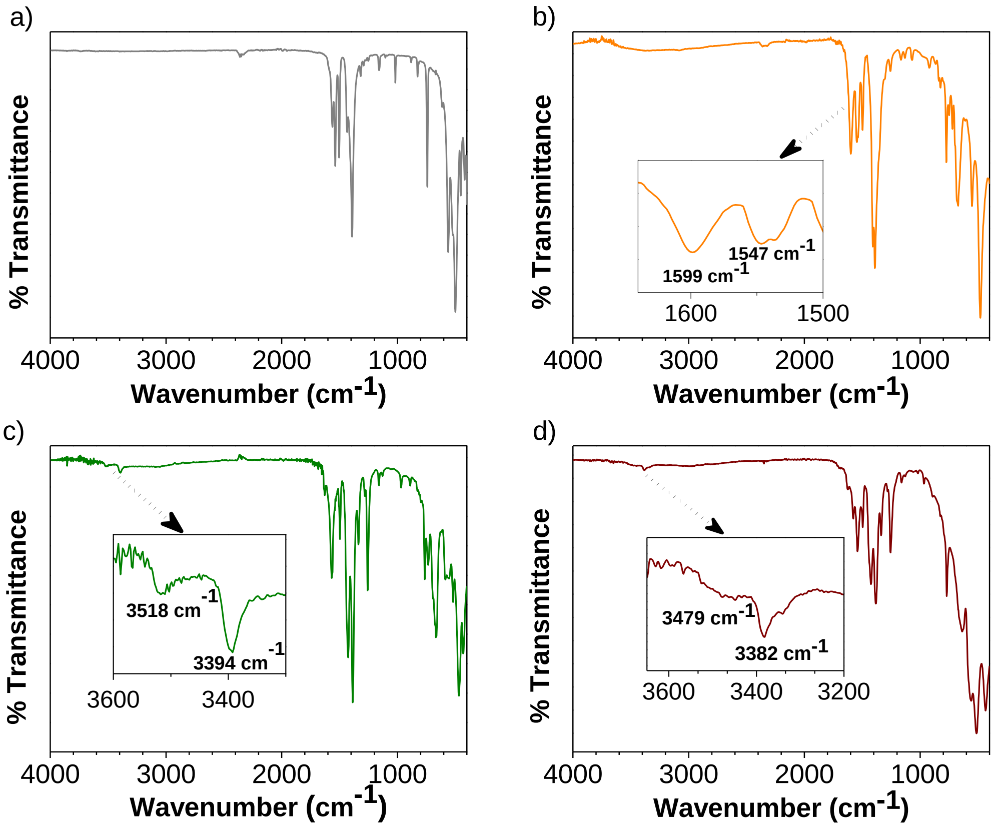

Figure S5. FT-IR spectroscopy of UiO-66(Zr) (**a**), UiO-66(Zr)-NO_2 (**b**), UiO-66(Zr)-NH_2 (**c**) and MIL-125(Ti)-NH_2 (**d**).

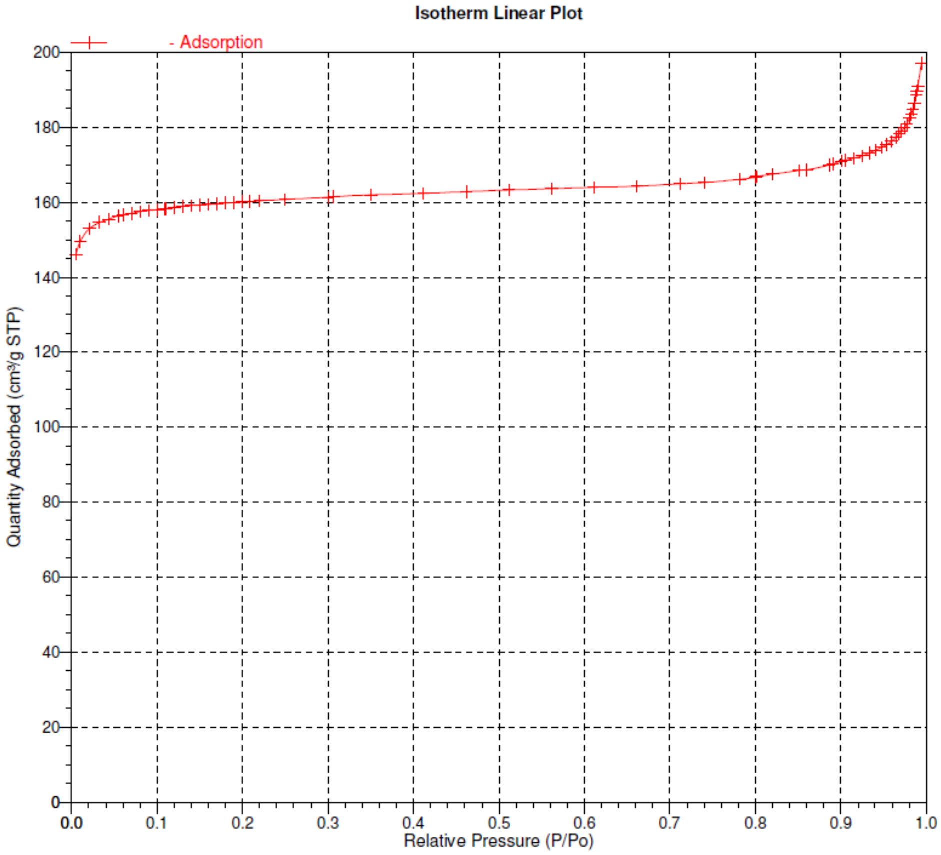

Figure S6. Isothermal N_2 adsorption curve of UiO-66(Zr).

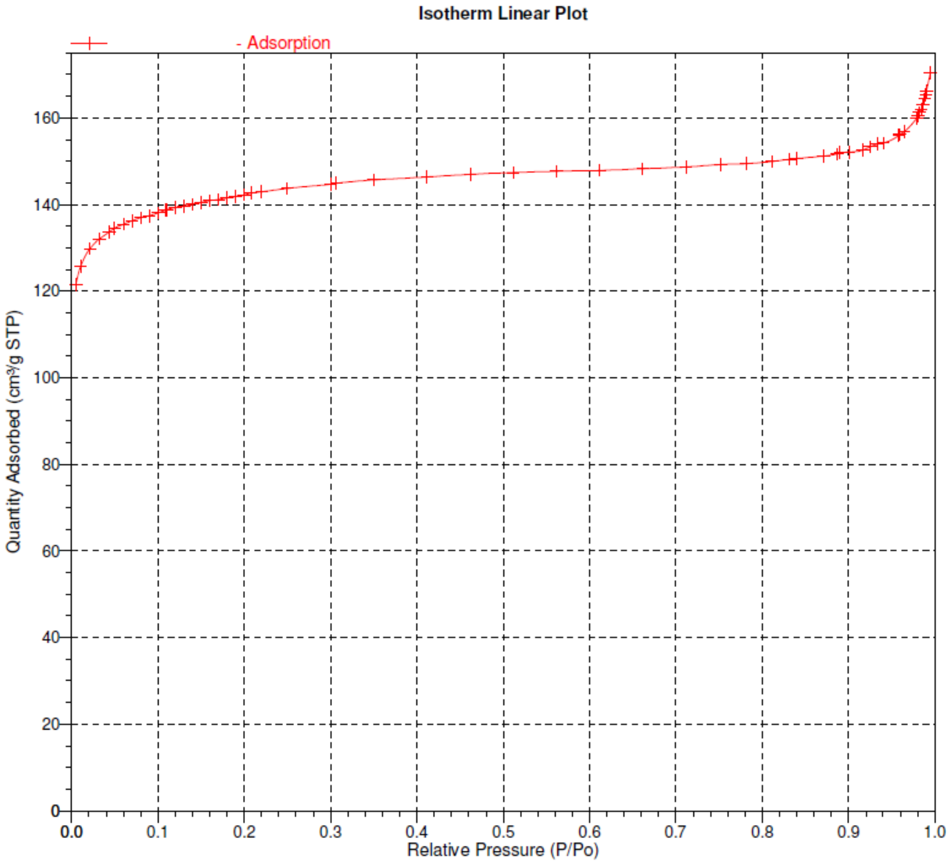

Figure S7. Isothermal N_2 adsorption curve of UiO-66(Zr)-NO_2.

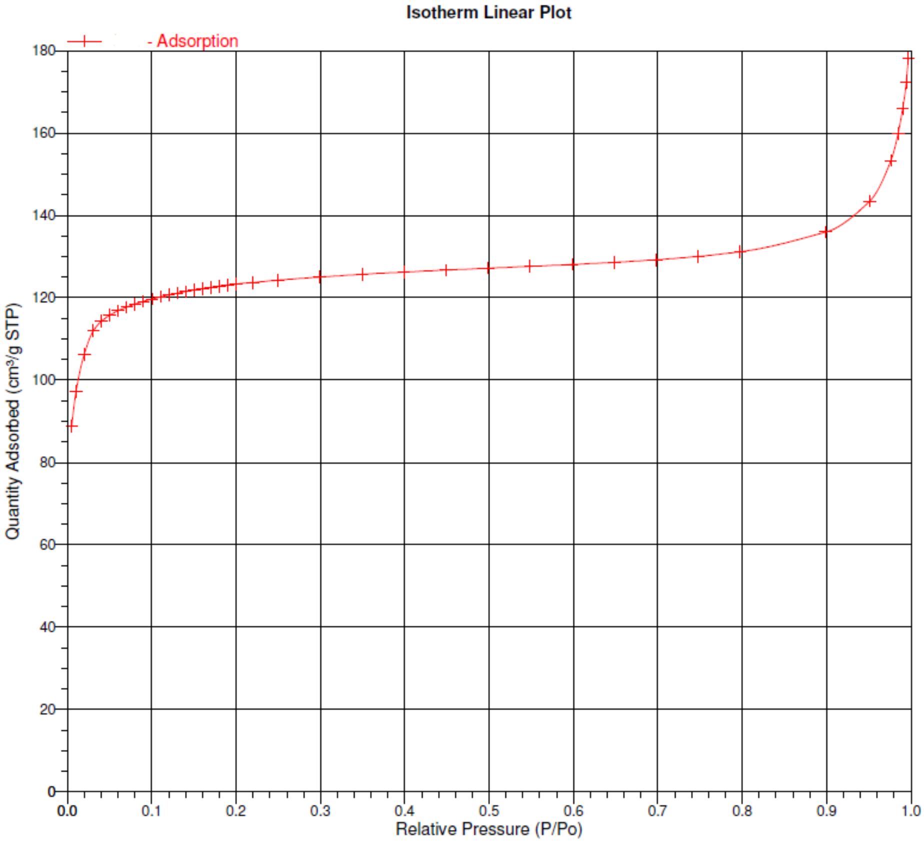

Figure S8. Isothermal N_2 adsorption curve of UiO-66(Zr)-NH_2.

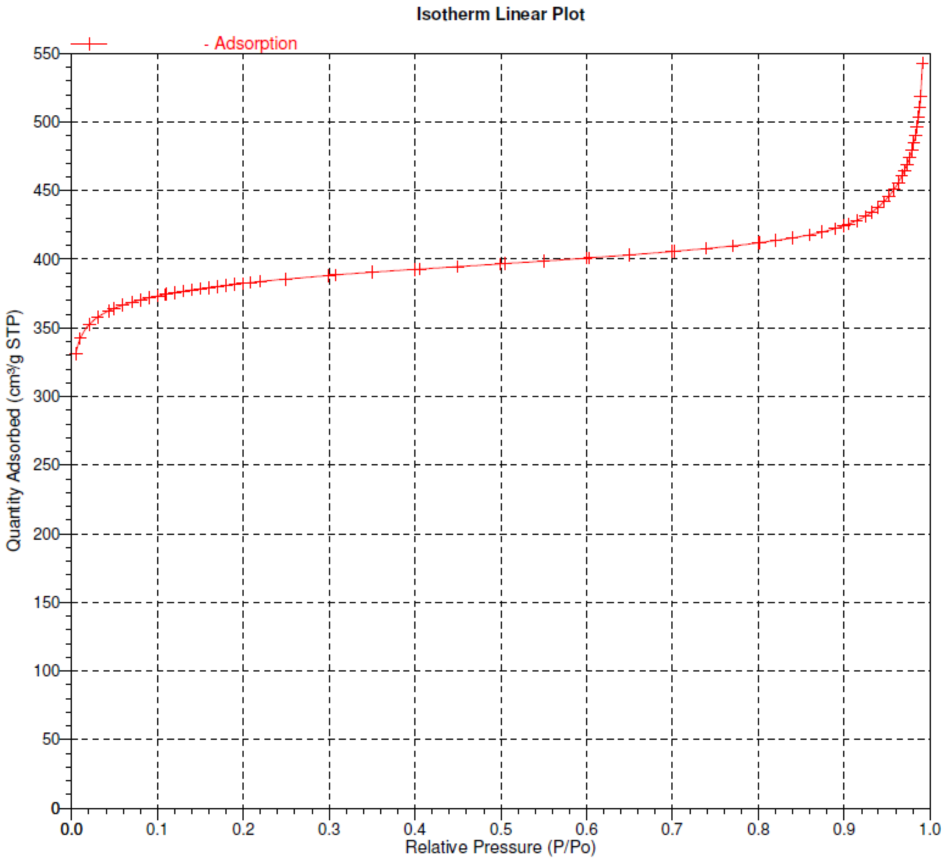

Figure S9. Isothermal N_2 adsorption curve of MIL-125(Ti)-NH_2.

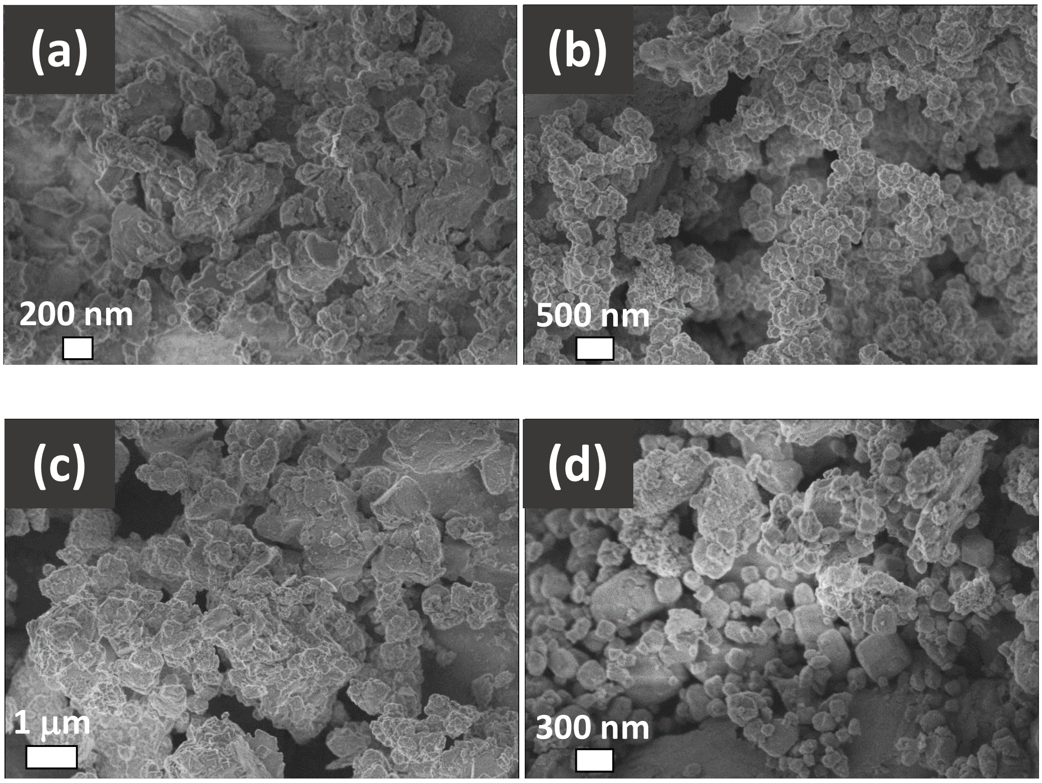

Figure S10. HR-SEM of (**a**) UiO-66(Zr)-H, (**b**) UiO-66(Zr)-NO_2, (**c**) UiO-66(Zr)-NH_2 and (**d**) MIL-125(Ti)-NH_2.

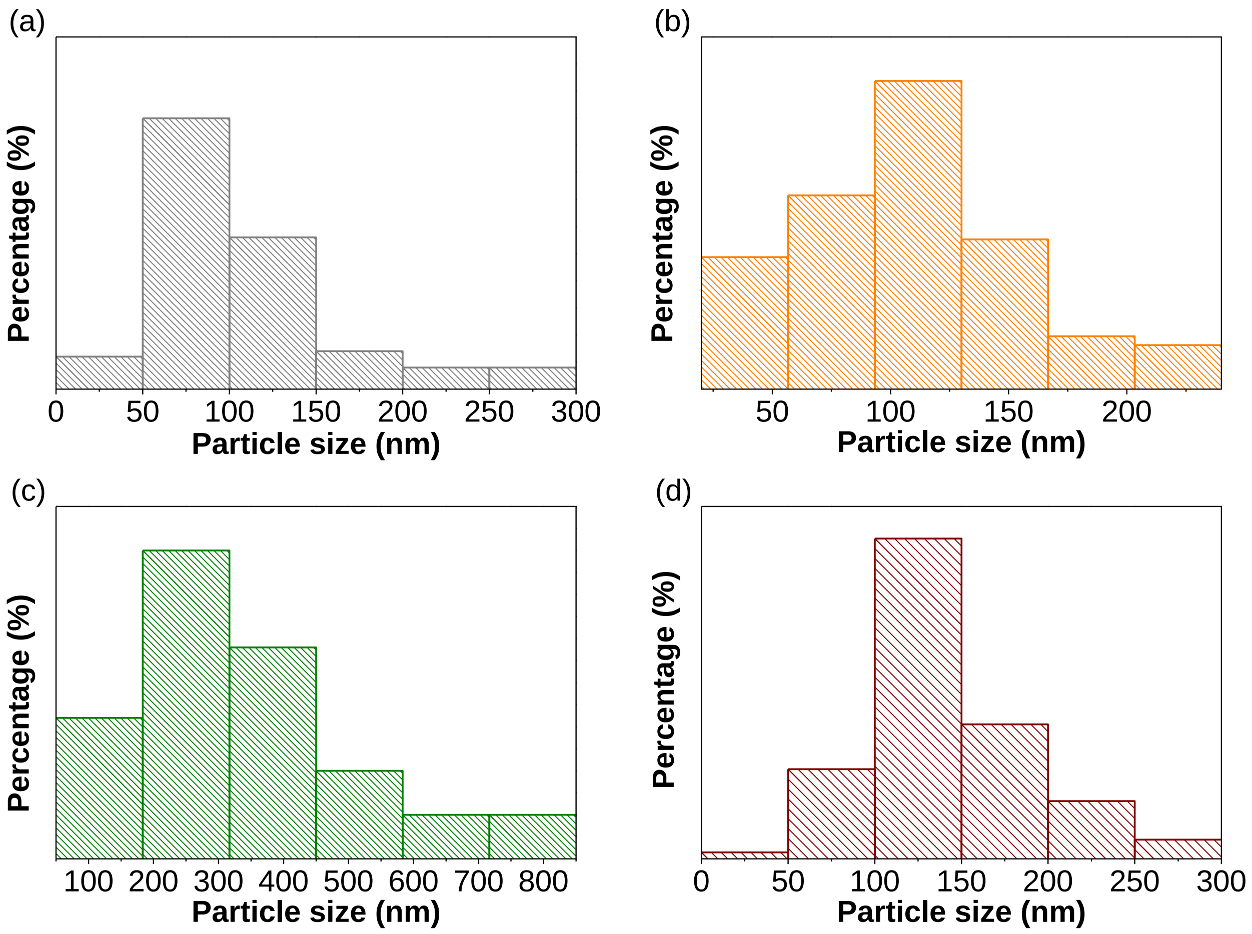

Figure S11. Particle size distribution of (**a**) UiO-66(Zr)-H, (**b**) UiO-66(Zr)-NO_2, (**c**) UiO-66(Zr)-NH_2 and (**d**) MIL-125(Ti)-NH_2.

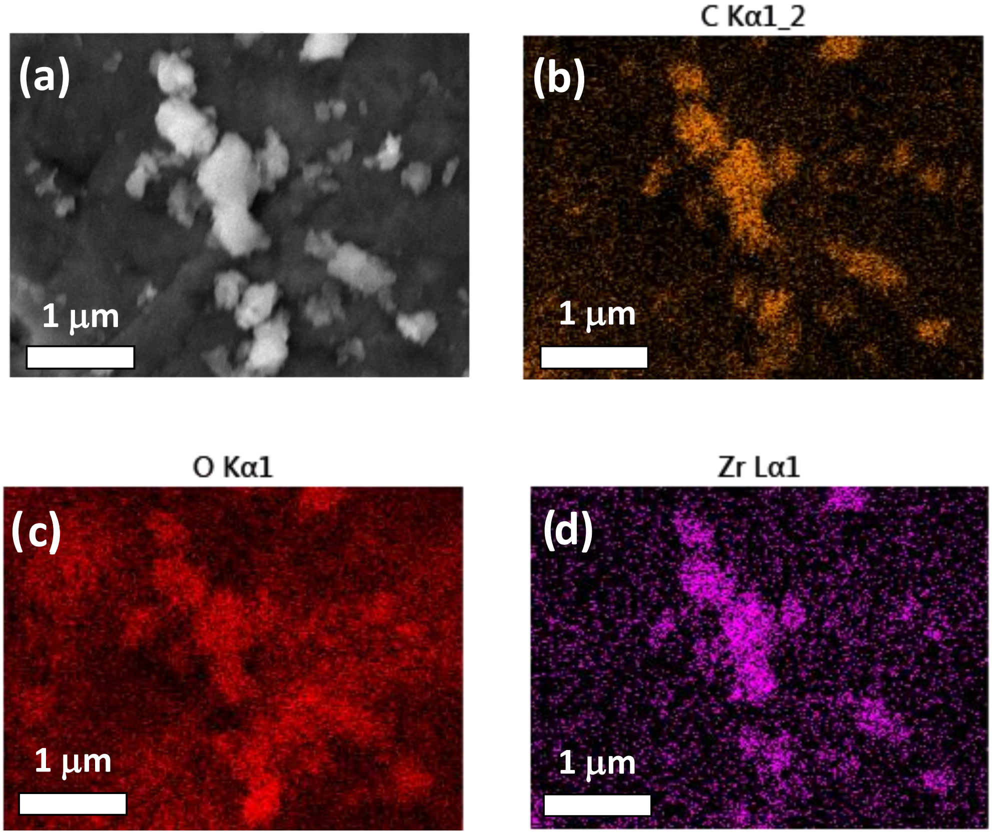

Figure S12. HR-SEM image of UiO66-(Zr) (**a**) and EDX mapping: carbon (**b**), oxygen(**c**), zirconium (**d**).

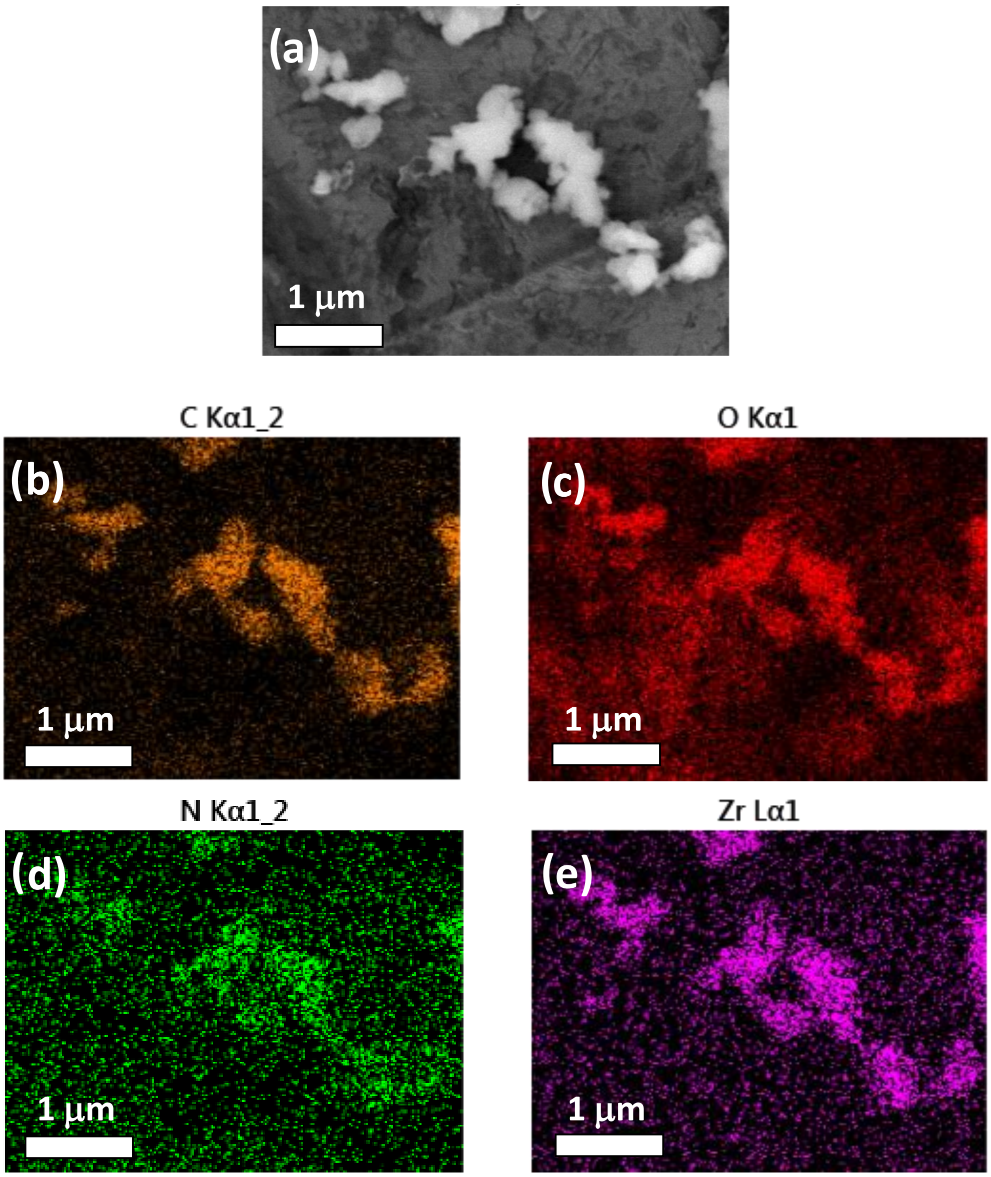

Figure S13. HR-SEM image of UiO66-(Zr)-NO_2 (**a**) and EDX mapping: carbon (**b**), oxygen(**c**), nitrogen (**d**), zirconium (**e**).

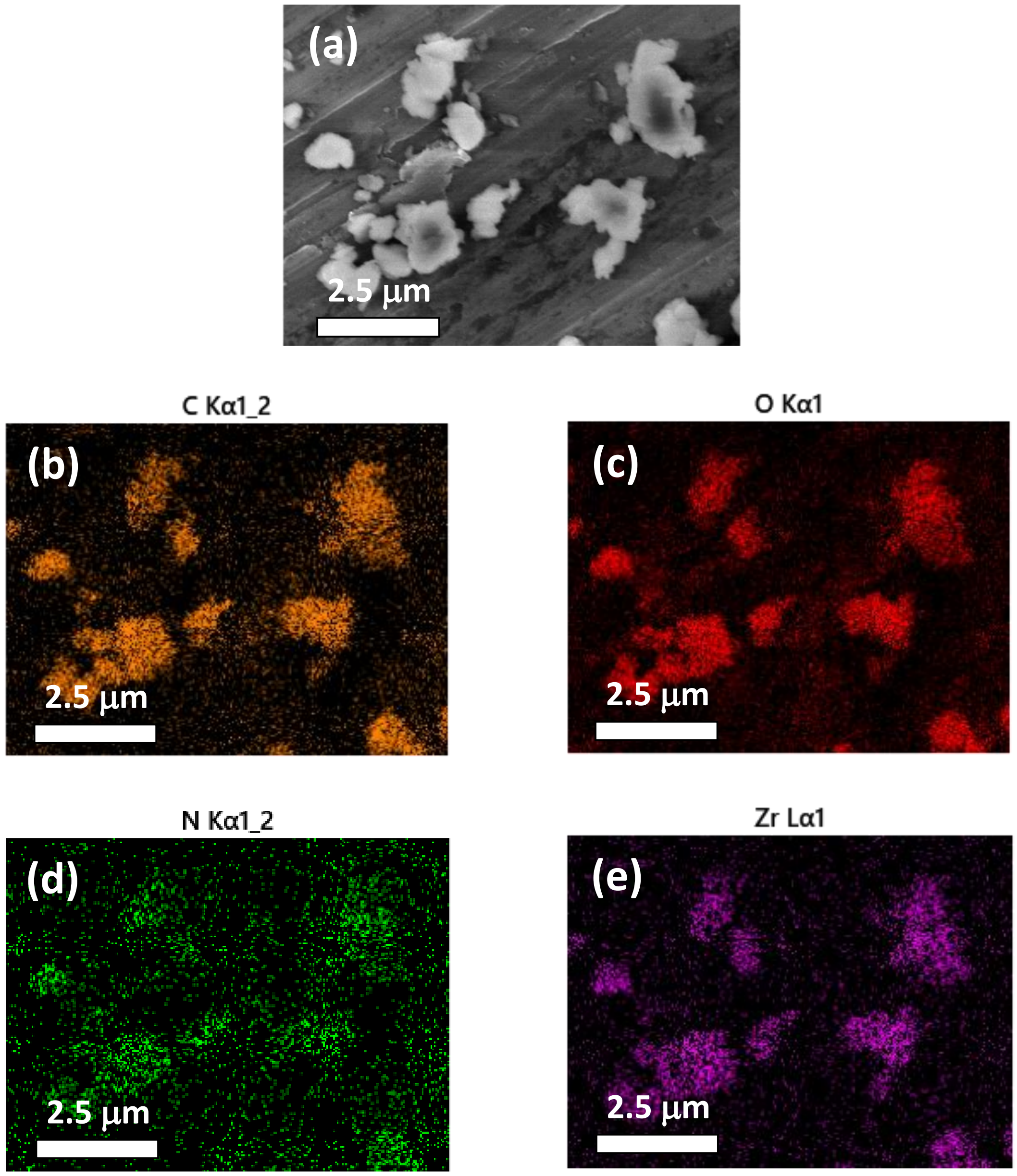

Figure S14. HR-SEM image of UiO66-(Zr)-NH_2 (**a**) and EDX mapping: carbon (**b**), oxygen(**c**), nitrogen (**d**), zirconium (**e**).

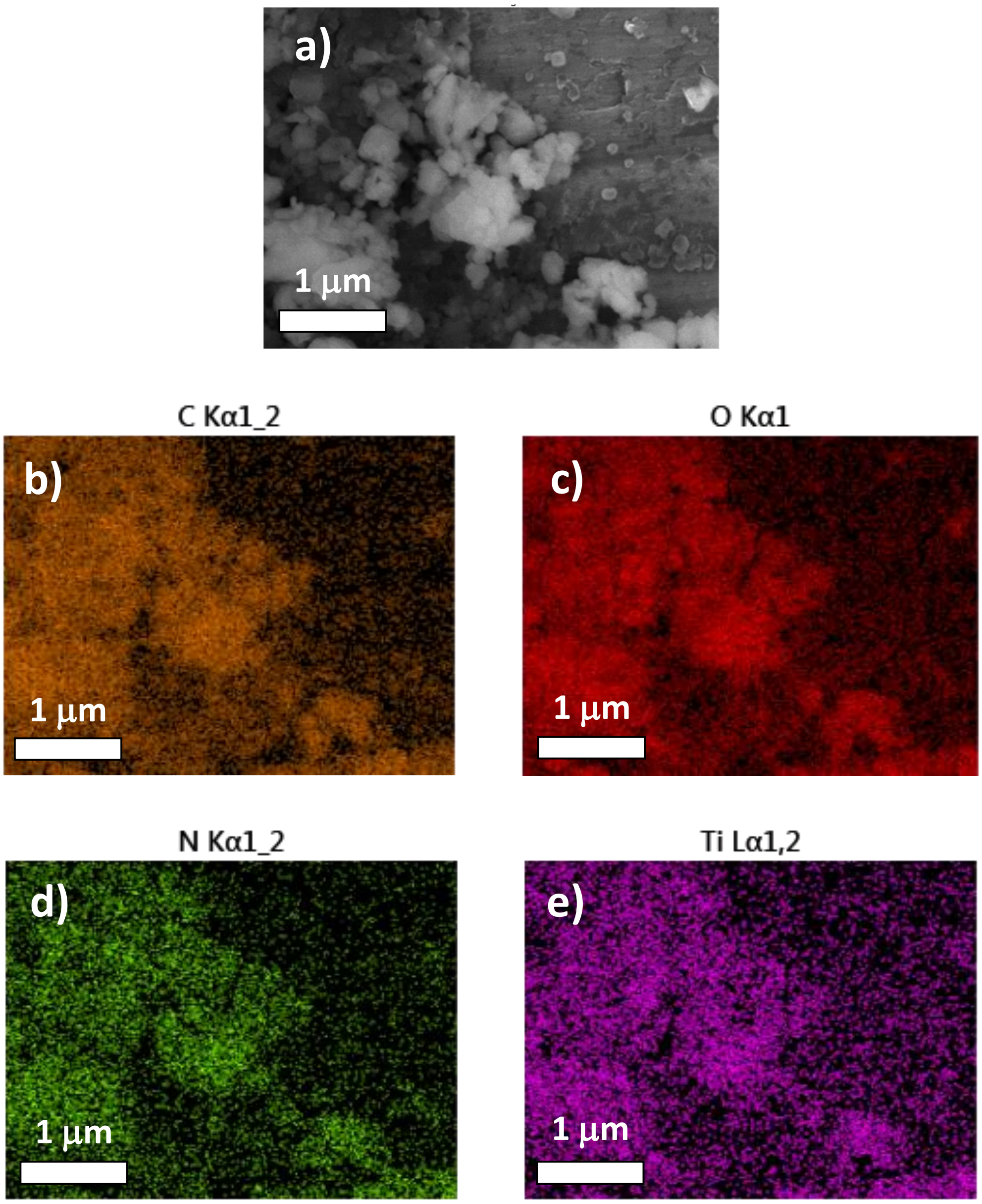

Figure S15. HR-SEM image of MIL-125-(Ti)-NH_2 (**a**) and EDX mapping: carbon (**b**), oxygen(**c**), nitrogen (**d**), titanium (**e**).

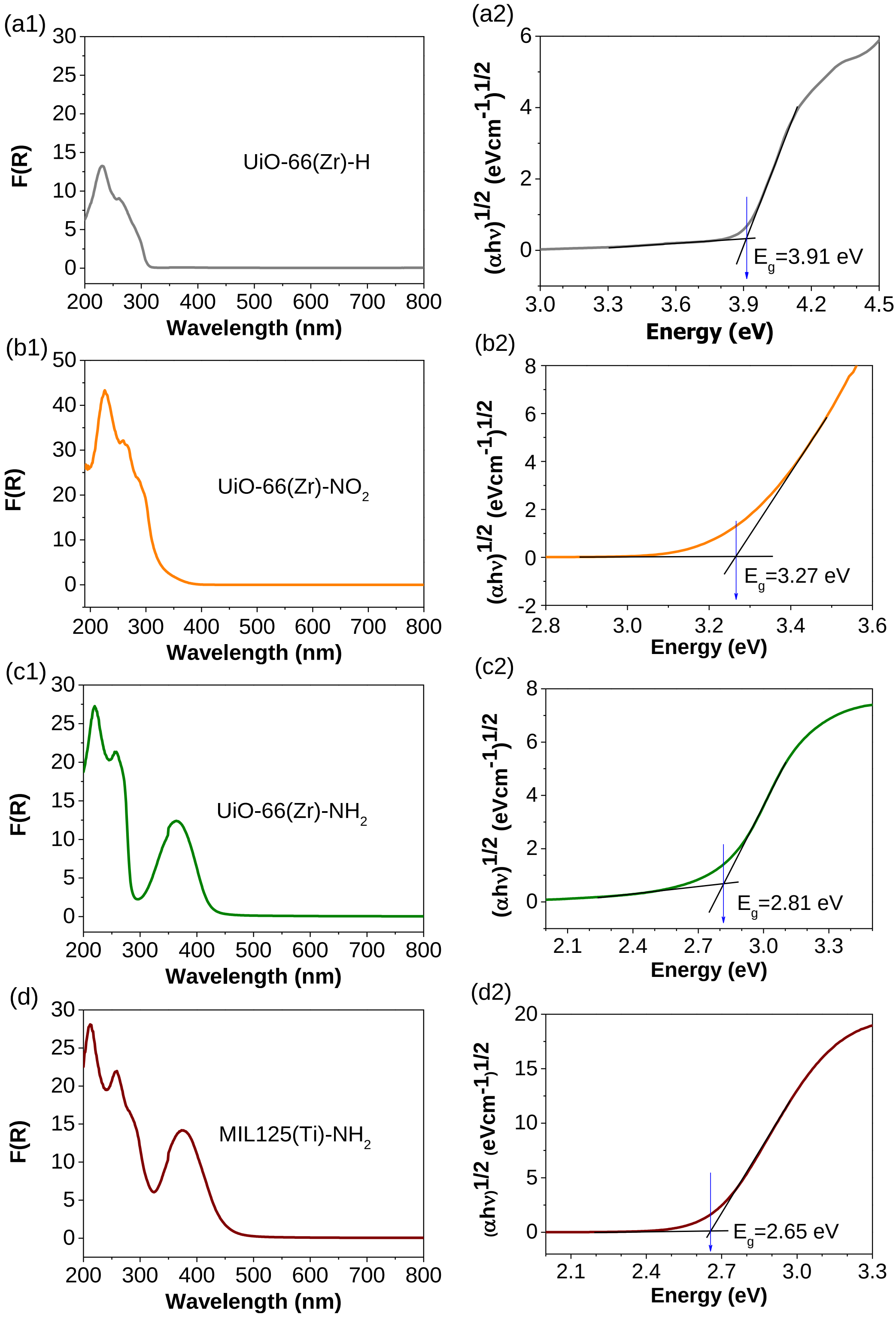

Figure S16. UV-Vis diffuse reflectance spectroscopy and its corresponding tauc plot: (**a**) UiO-66(Zr)-H, (**b**) UiO-66(Zr)-NO_2, (**c**) UiO-66(Zr)-NH_2 and (**d**) MIL-125(Ti)-NH_2.

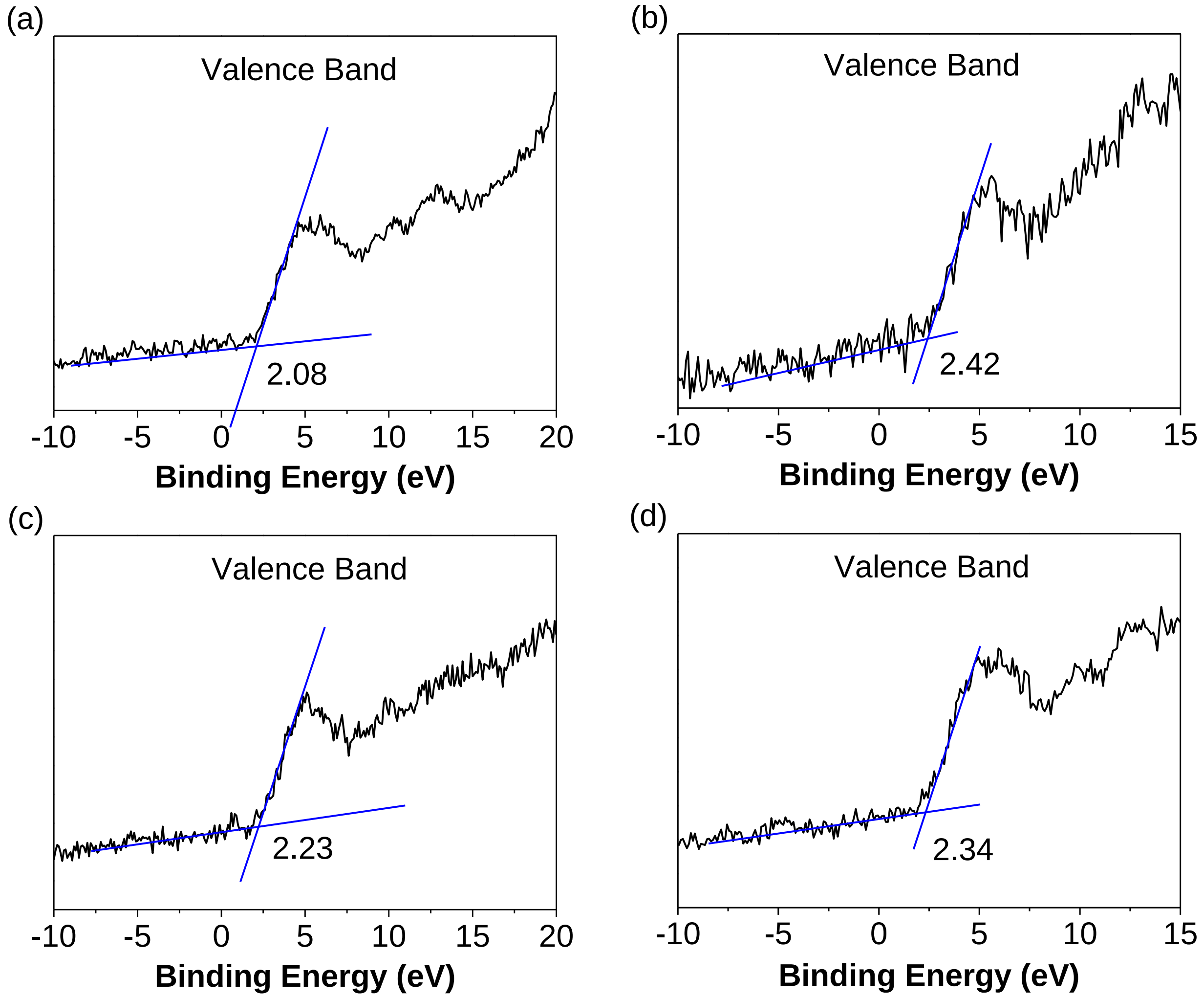

Figure S17. Valence band of (**a**) UiO-66(Zr)-H, (**b**) UiO-66(Zr)-NO_2, (**c**) UiO-66(Zr)-NH_2 and (**d**) MIL-125(Ti)-NH_2.

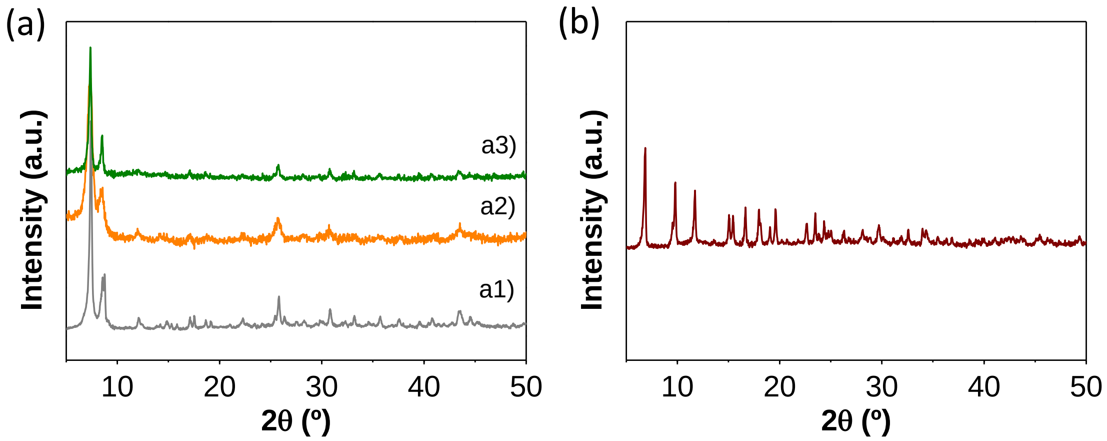

Figure S18. (**a**) XRD patterns of used UiO-66(Zr) (a1), UiO-66(Zr)-NO_2 (a2) and UiO-66(Zr)-NH_2 (a3). (**b**) XRD patterns of used MIL-125(Ti)-NH_2.

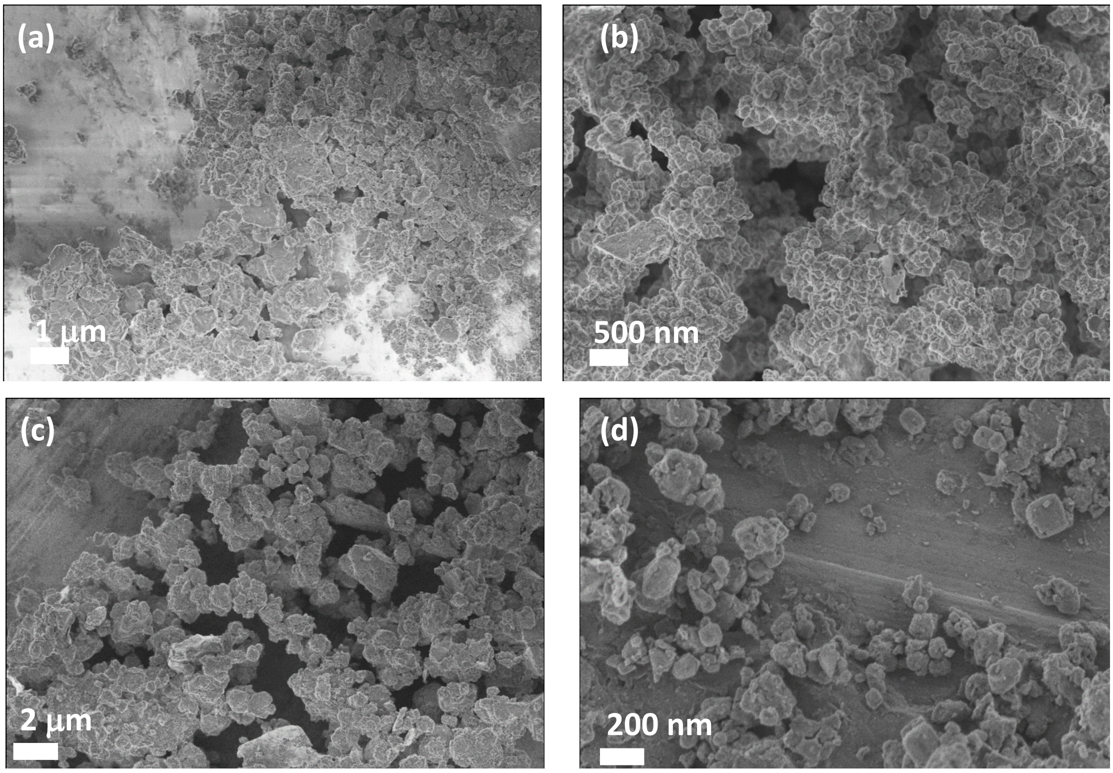

Figure S19. HR-SEM of used (**a**) UiO-66(Zr)-H, (**b**) UiO-66(Zr)-NO_2, (**c**) UiO-66(Zr)-NH_2 and (**d**) MIL-125(Ti)-NH_2.

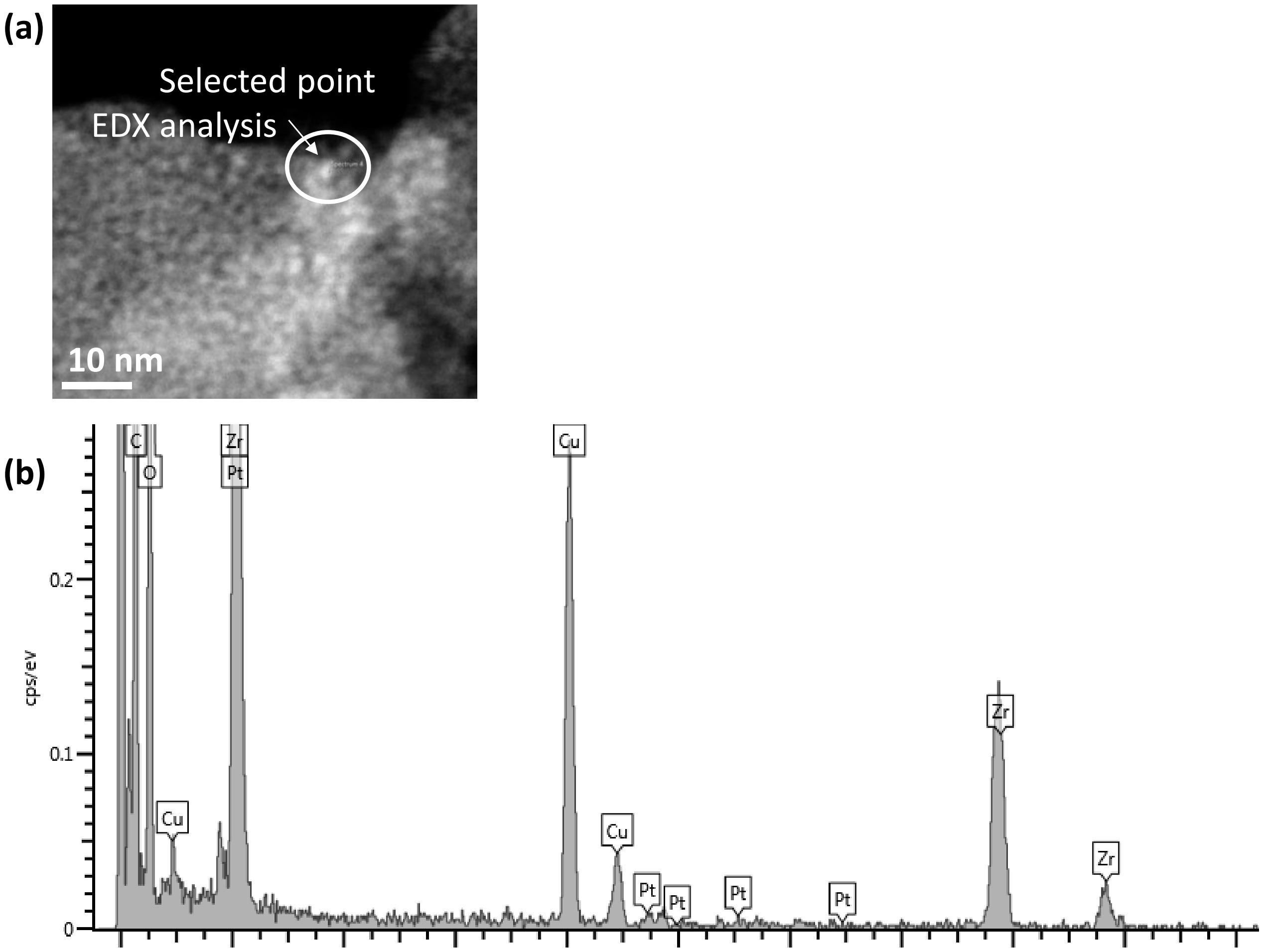

Figure S20. Representative DF-STEM image (**a**) and point EDX analysis of fresh Pt/UiO-66(Zr)-NH_2 (**b**).

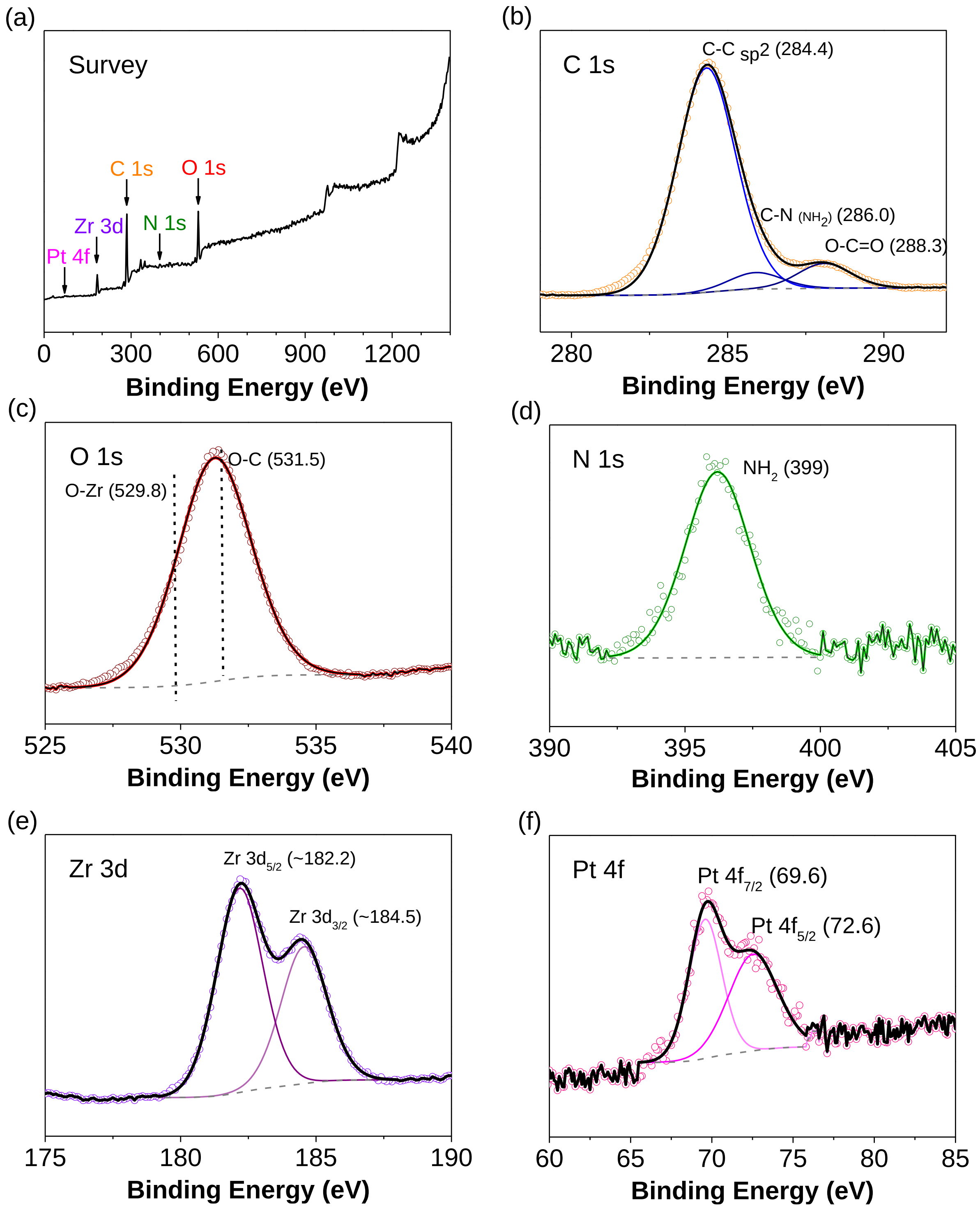

Figure S21. (**a**) XPS C 1s (**b**), O 1s (**c**), N 1s (**d**), Zr 3d (**e**) and Pt 4f (**f**) of 1%wtPt@UiO-66(Zr)-NH_2.

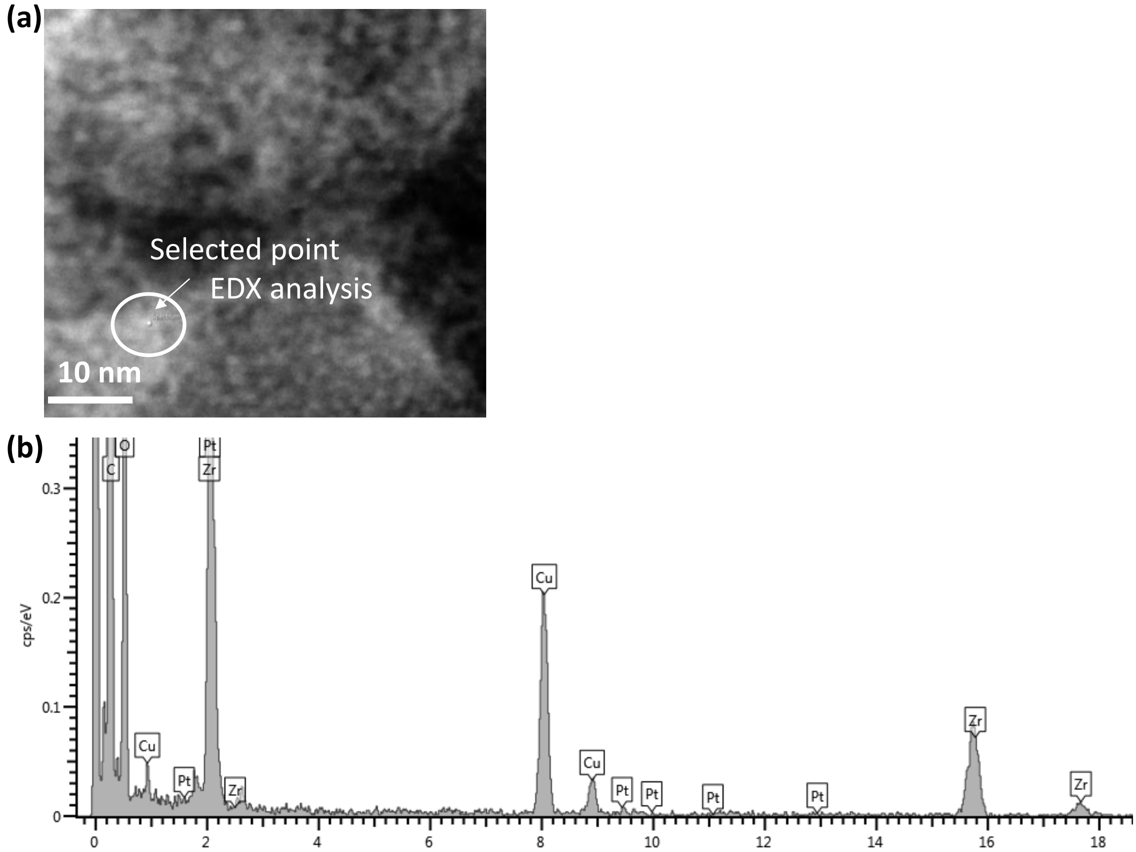

Figure S22. Representative DF-STEM image (**a**) and point EDX analysis of the Pt/UiO-66(Zr)-NH_2 photocatalyst used for 70 h (**b**).

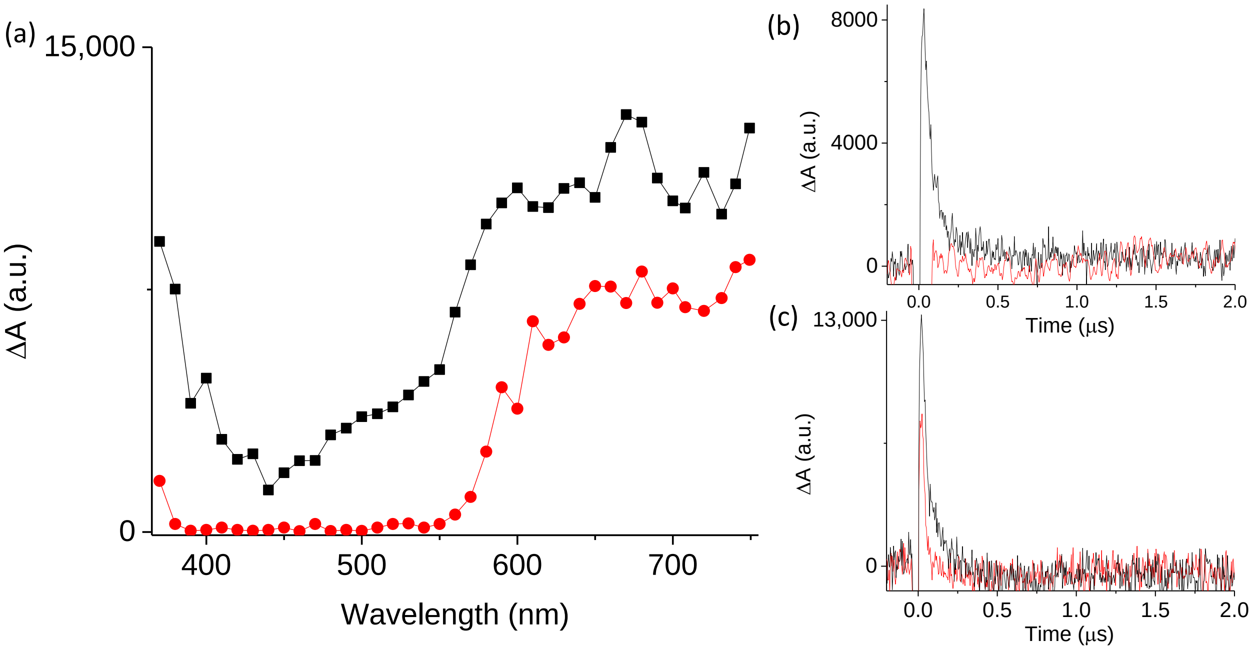

Figure S23. TAS spectra of Pt@UiO-66(Zr)-NH_2 purged with argon (■) and after adding methanol (●) (**a**). Absorbance transition decay of Pt@UiO-66(Zr)-NH_2 before (black line) and after adding methanol (red line) recorded at 380 nm (**b**) and 680 nm (**c**).

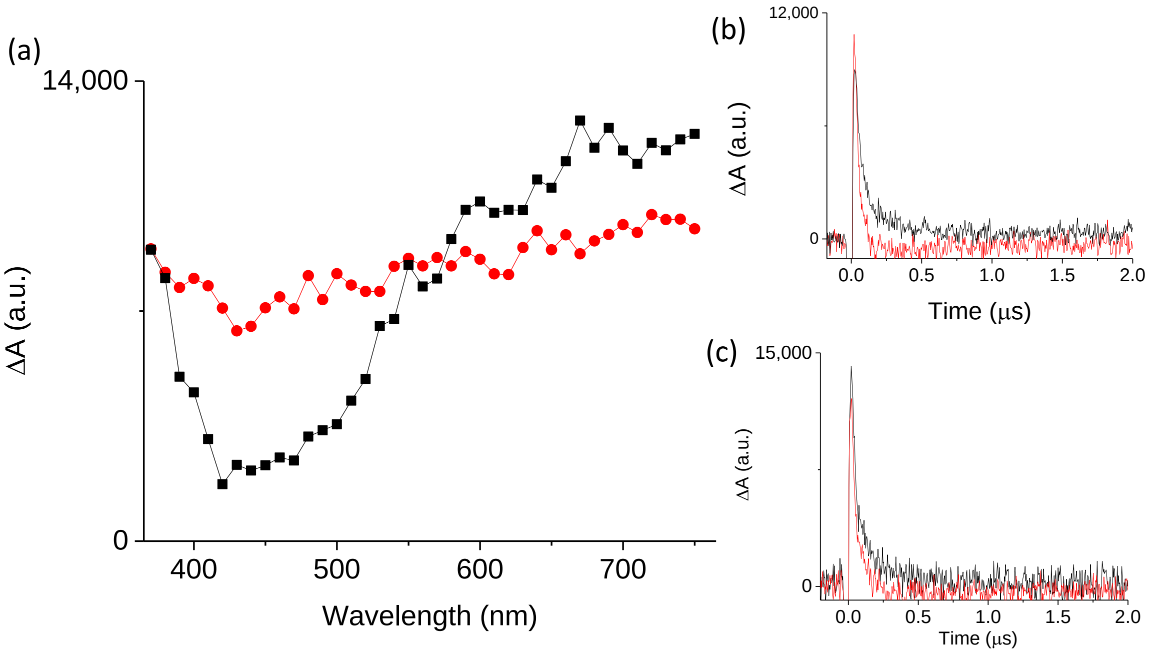

Figure S24. TAS spectra of Pt@UiO-66(Zr)-NH_2 purged with argon (■) and after adding molecular oxygen (●) (**a**). Absorbance transition decay of Pt@UiO-66(Zr)-NH_2 before (black line) and after adding molecular oxygen (red line) recorded at 380 nm (**b**) and 680 nm (**c**).

References

1. Melillo, A.; García-Vallés, C.; Ferrer, B.; Álvaro, M.; Navalón, S.; García, H., Bifunctional Metal-Organic Frameworks for Hydrogenation of Nitrophenol using Methanol as Hydrogen Source. *Org. Biomol. Chem.* **2021,** *19*, 794-800.
2. Shearer, G. C.; Chavan, S.; Bordiga, S.; Svelle, S.; Olsbye, U.; Lillerud, K. P., Defect Engineering: Tuning the Porosity and Composition of the Metal−Organic Framework UiO-66 via Modulated Synthesis. *Chem. Mater.* **2016,** *28*, 3749−3761.
